# Author Correction: The *Aphelenchus avenae* genome highlights evolutionary adaptation to desiccation

**DOI:** 10.1038/s42003-021-02824-5

**Published:** 2021-11-12

**Authors:** Xuehua Wan, Jennifer A. Saito, Shaobin Hou, Scott M. Geib, Anton Yuryev, Lynne M. Higa, Christopher Z. Womersley, Maqsudul Alam

**Affiliations:** 1 Advanced Studies in Genomics, Proteomics and Bioinformatics, University of Hawaii, Honolulu, HI USA; 2 TEDA Institute of Biological Sciences and Biotechnology, Nankai University, Tianjin, P. R. China; 3 Tropical Crop and Commodity Protection Research Unit, USDA-ARS Pacific Basin Agricultural Research Center, Hilo, HI USA; 4 Elsevier Life Sciences Solutions, Rockville, MD USA; 5 School of Life Sciences, University of Hawaii, Honolulu, HI USA

**Keywords:** Evolution, Computational biology and bioinformatics, Genetics

Correction to: *Communications Biology* 10.1038/s42003-021-02778-8, published online 28 October 2021.

The original version of this Article omitted the following statement in the Acknowledgements: “USDA is an equal opportunity employer. Mention of trade names or commercial products in this publication is solely for the purpose of providing specific information and does not imply recommendation or endorsement by the USDA.” This has been corrected in both the PDF and HTML versions of the Article.

