# Supplementary material for: The Aphelenchus avenae genome highlights evolutionary adaptation to desiccation
Source: Commun Biol. 2021 Oct 28;4:1232. doi: 10.1038/s42003-021-02778-8 (PMC8553787; doi:10.1038/s42003-021-02778-8)
Supplement: Supplementary file 2 — Supplementary Information [file 42003_2021_2778_MOESM2_ESM.pdf]

SUPPLEMENTARY NOTE

1. Genome size estimation

Flow cytometry analysis showed a primary peak at G0 + G1 phase of cell cycle, suggesting that *A. avenae* nuclei has a diploid genome. The average C-value was estimated to be 0.52 and the standard deviation is 0.005, indicating that *A. avenae* genome size is 255.32 Mb and the standard deviation is 2.41 Mb, based on the below equation [1].

Genome size (bp) = 0.978 x 10^9 x DNA content (pg) (1)

Besides relying on flow cytometry analysis, we counted occurrences of 31-mers of Illumina reads and 454 reads to estimate the genome size (**Supplementary Figure 1**). The probabilities of 31-mer frequencies were plotted. The frequency distribution peak of all the 31-mers was 42. The low frequency of the 31-mers was caused by sequencing errors, while the high frequency of the 31-mers was due to repeat regions in the genome. Thus, those 31-mers counted near the peak represented the non-repeat regions of the genome. The curve with 31-mer frequencies from 30 to 48 fitted gamma distribution by least squares fitting method (Pearson's r = 0.99). The total number of the 31-mers with frequencies from 30 to 48 was divided by multiplicity of the 31-mer peak to estimate the non-repeat region of the genome above ~206 Mb. The total number of the 31-mers with frequencies from 28 to 51 was divided by multiplicity of the 31-mer peak to

estimate the genome size above ~255 Mb (Pearson's r = 0.98). Pearson's correlation coefficient was calculated by R package Hmisc v3.10.

2. Genome assembly and validation

The statistics of Illumina and 454 reads were listed in **Supplementary Table 1**. To assemble the long 454 reads and short Illumina reads with high coverage, we used two different strategies: 1) We normalized the 163 X Illumina reads to 30 X *in silico* by Trinity v2.0.5[1], and then loaded the normalized Illumina paired-end reads with 454 shotgun, 8-kb and 20-kb paired-end reads to newbler 2.8 (Roche, USA) for assembly; 2) We assembled Illumina reads using SOAPdenovo v2[2]. By setting 47-mer as K value, 424,453,084 nodes were allocated, and 20,025,235,537 of 47-mers were generated. A total of 42,677 scaffolds had been built to contain 536,819 contigs and show a ~ 292 Mb span. After *in silico* gap closing, 231,941 gaps were closed. The longest scaffold contained 154,661 bp. The average scaffold length was 6,841 bp, and the scaffold N_{50} length was 12,617 bp. The consensus contigs were splitted to 400 bp pseudo reads with 200 bp overlaps, and then these 1,491,907 pseudo reads were assembled with 454 shotgun reads, 8-kb and 20-kb paired-end reads together using newbler 2.8. We set newbler parameters as Large or Complex Genome (-large), Heterozygotic Mode (-het), and Gap Filling Mode (-scaffold). The large contig threshold and scaffold length threshold were set as 500 bp and 2,000 bp separately. A total of 18,660 scaffolds were built. The largest scaffold length was 5.5 Mb, and scaffold N_{50} was ~ 142 kb. The assembly contained 34,992 contigs with ~ 191 Mb consensus bases. The largest scaffold

contig size was 139 kb and the contig N_{50} length was 6.8 kb. The ~ 554 million paired-end Illumina reads were used to close gaps in scaffolds. After gap closing, 3,317 gaps were closed, and the span of consensus bases was increased to ~ 223 Mb. The largest contig length was ~153 kb, and the contig N_{50} length was ~ 11 kb.

The assembly generated a total of 289,186 singleton reads, of which 284,182 (98.3%) singletons were 454 reads and 5,004 (1.7%) were pseudo reads. The singleton pseudo reads were only 0.3% of the total pseudo reads used for newbler assembly, indicating that 99.7% of pseudo reads were successfully assembled by newbler. The G + C content distribution of 454 singleton reads was plotted and its peak shifted left compared to that of assembled scaffolds. About 30% of 454 singleton reads were under 200 bp and 12.7% were identified as repeats. Roche 454 singleton reads were used as query to search against NCBI nucleotide collection (nt) database by BLASTN (e value cutoff = $1e^{-5}$) and the BLASTN output was loaded to MEGAN v5.5.3 program to perform taxonomic analysis. A total of 89,472 singleton sequences (31.5%) had no hits, 24,005 singleton sequences (8.4%) were assigned as Bacteria, and 3,867 singleton sequences (1.4%) were assigned as Eukaryota, suggesting that most *A. avenae* reads were assembled into the scaffolds.

The paired-end Illumina reads were re-aligned to the assembled scaffolds to evaluate the coverage by both the SOAPaligner-SOAPcoverage package and bowtie2-SAMtools package. The coverage distributions generated by SOAPcoverage were plotted in **Supplementary Figure 2**. SOAP package generated the average coverage as ~ 125X, while bowtie2 v2.1.0 and SAMtools v1.1 generated the average coverage as ~ 160X.

The difference of calculated coverages came from alignment methods, since SOAP package performed un-gapped alignments but bowtie2 fully supported gapped alignments with affine gap penalties.

To exclude DNA contaminations from *Rhizoctonia solani*, we screened the assembled *A. avenae* genome against sixteen current available genomes of *R. solani* that were downloaded from the NCBI database. The GenBank accession numbers of the sixteen *R. solani* genomes included GCA_016906535.1, GCA_017311305.1, GCA_015342405.1, GCA_015342435.1, GCA_015341985.1, GCA_015342415.1, GCA_000715385.1, GCA_000832345.2, GCA_000524645.1, GCA_000334115.1, GCA_001286725.1, GCA_900185085.1, GCA_000695385.1, GCA_001899475.2, GCA_000350255.1, GCA_003268435.1. BLASTN searches (E_value ≤ 1e-5) identified ~ 180 average hits per *R. solani* genome (ranged from 23 hits to 264 hits). The Hits with aligned lengths above 1kb were identified as 18S ribosomal RNA homologs (percentages of identical matches: 75.6% ~ 77.8%). The hits with aligned lengths ranged from 100 bp to 600 bp were identified as parts of 18S, 28S ribosomal RNA homologs or highly conserved mRNA sequences that were also found in other species by manual BLASTN searches at the NCBI website. The majority of the hits showed less than 90 bp (percentages of identical matches < 100%). These data confirm that there are no contaminations from *R. solani* DNA sequences in the assembled *A. avenae* genome.

To evaluate the completeness of the assembly, we used a core eukaryotic genes mapping approach (CEGMA v2.5)[3]. A collection of 2,748 core eukaryotic genes were

used to identify homologs in *A. avenae* genome by combining 4 programs including geneid, GeneWise, HMMER, and BLAST+. The 2,748 protein sequences were mapped to *A. avenae* genome by TBLASTN first. Then GeneWise and geneid were used to predict core genes initially. The predicted proteins were filtered by HMMER. Geneid parameters were calculated from selected geneid predictions, and then the calculated geneid parameters were used for accurate mapping. HMM was used for final filtering and identification. For BUSCO-based evaluation, we carried out BUSCO v5.0.0 with metaeuk and obtained 92.6%, 77%, and 73.6% of the BUSCO orthologs in the assembly using eukaryote_odb10 (255 genes, 70 species), metazoan_obd10 (954 genes, 65 species) and nematode_obd10 (3,131 genes, 7 species) datasets, respectively.

3. Single nucleotide polymorphisms (SNPs)

A. avenae contains a diploid genome based on flow cytometry analysis. *A. avenae* can reproduce either sexually or asexually. However, the occurrence of males was very low[4], and *A. avenae* mainly reproduces by parthenogenesis. Thus, SNP dataset of a monoxenic population will be expected to be close to that of a diploid individual. In addition, the various base callings can be introduced by sequencing errors. To avoid sequencing artifacts, the Illumina paired-end reads were trimmed by using Phred quality score 30 (Q30) as quality control, which generated a set of reads with above 99.9% accuracy. We generated 567 million of paired-end reads using Q30 trimming. Illumina reads were mapped to the assembled scaffolds by gapped-alignment software bowtie2. About 79% of Illumina paired-end reads were aligned to the draft assembly. Heterozygous SNPs were called by SAMtools[5] and bcftools. The parameter -D option

of varFilter for vcfutil command was set to 320 which was about twice of the average mapped read depth, in order to filter out repetitive sites. Transitions and transversions were counted by vcftools. A total of 729,985 SNP sites were identified. The mean SNP density was about 3.13 variants per kb. There were 400,474 transition sites and 266,268 transversion sites. Ts/Tv ratio was ~1.5.

4. Protein coding gene and comparative annotation

A total of 43,192 gene models were identified by Evidence Modeler (EVM) v1.1.1. While 35,554 gene models (82.3%) had stop codon, 41,134 gene models (95.2%) started with amino acid methionine. The 17.7% fragmented gene models were caused by segmentation of the draft assembled scaffolds. About 34,357 gene models (~ 80%) had expression values in transcriptome analysis. The results of Pfam (v30.0) annotation were listed in **Supplementary Data 1**. Pfam domain comparisons among *A. avenae*, *C. elegans*, fruit fly, Sea Urchin and human were listed in **Supplementary Table 4**. Gene ontology was carried out using WEGO[6, 7].

To identify the orthologous genes and species-specific genes between *A. avenae* and clade IV species, we downloaded protein sequences of *Steinernema carpocapsae*[8], *Meloidogyne hapla*[9], *Globodera pallida*[10], *Meloidogyne incognita* (population Morelos) [11], *Meloidogyne incognita* (W1 strain)[12], *Bursaphelenchus xylophilus*[13] and *Panagrolaimus superbus*[14] from WormBase ParaSite website (https://parasite.wormbase.org/index.html, version: WBPS15 (WS276)). OrthoFinder v2.3.9[15,16] employing MCL clustering algorithm was carried out for ortholog inference.

The comparison results were listed in **Supplementary Table 7**. Both *A. avenae* (47.7%) and *S. carpocapsae* (50%) showed high percentages of species-specific genes (singletons and genes in lineage-specific gene families), indicating that the genomes of nematodes, even from the same clade, are highly variable in gene contents.

5. Operon

In bacteria, genes are organized in operons to adapt quickly to environment changes. Though unusual, gene clusters sharing the same promoter were observed in eukaryotes, evidenced in *Trypanosome*, nematode *C. elegans*, and chordates[17-19]. Genomic analysis suggested that 17% of *C. elegans* protein-coding genes may be organized into operons[20]. *C. elegans* and *B. malayi* (WormBase WS236 releases) contained 1,390 and 1,535 operons with 3,433 (16.7%) and 3,800 (26.7%) gene models respectively. To identify potential operons in *A. avenae*, we counted genes which located on the same strand and had intergenetic regions ranging from 25 ~ 1,000 bp. We predicted 4,453 operons containing 10,063 gene models (23.3%) (**Supplementary Data 2**). Out of these 10,063 operon containing genes, 1,782 gene models (17.7%) were singletons and 6,141 gene models (61%) were duplicated by dispersed duplication.

6. Molecular shield

6.1. Novel intrinsically disordered protein (IDP)

By manually annotating the top 30 significantly up-regulated proteins in desiccation, we found that 21 (70%, higher than the average 44%) proteins were novel proteins with unknown functions. Based on protein disorder database improved on long disordered

regions, ten of the twenty-one novel proteins are either fully disordered or with only a few ordered amino acids. These proteins are predicted to be able to form alpha-helical structures, thus may have a chaperone like function as LEA and anhydrin during desiccation stress. We further collected the sequences of novel up-regulated proteins which have no hit by BLASTP searches against NCBI nr database and examined those intrinsically disordered regions. Intrinsically disordered regions of proteins were calculated by IUPred[21], which recognized intrinsically disordered regions based on the estimated pairwise energy content. Scores above 0.5 indicated disorder. We identified 137 IDPs (**Supplementary Data 4**). Species-specific IDP were determined by having no orthologs in any of the other seven nematode species (**Supplementary Data 5**).

6.2. Late embryo abundant (LEA) proteins

Most of late embryogenesis abundant (LEA) proteins, which are reported to protect other proteins from aggregation during desiccation or osmotic stress[22], are well characterized examples as IDPs in plants, bacteria and some invertebrates[23]. They are extremely hydrophilic and largely unstructured, but group 3 LEA proteins contain an 11-amino acid motif and can potentially fold into alpha-helical structures during drying process[22,24]. Identification and characterization of single group 3 LEA protein in *A. avenae* were previously reported[25,26]. Eight LEA group 3 protein sequences of nematodes and plants (**Supplementary Table 8**) were collected from NCBI database and used as input query to search for homologous LEA group 3 proteins in *A. avenae*. Fifteen LEA group 3 proteins were identified by BLASTP (E_value < 1e-4) (**Supplementary Table 9**). Multiple protein sequence alignment was performed by

MAFFT v7.046b. Phylogenetic tree was generated by ClustalW2 v2.1[27] and displayed by EvolView v1[28] (**Figure 3b**). LEA sequence logo was identified by WebLogo v2.8 program (http://weblogo.berkeley.edu/) (**Supplementary Figure 23**). The highly hydrophilic motif "E/D-x-x-K/Q-E/D-K-x-x-E/N/D/S-x-x-E/Q-x-TKE/D" is predicted to be able to form helical structure by an HMM algorithm. To search for remote homologs, we used an HMM-HMM alignment comparison method[29,30] and found that the most alike structure of the *A. avenae* LEA conserved motif is human micelle-bound alpha-synuclein (90% probability), which is the non-Abeta component of Alzheimer's disease amyloid found in neural tissue and is an unstructured soluble protein but forms alpha-helical structure when binds to hydrophobic lipid membranes[31,32]. Thus LEA may obtain a functional alpha-helical structure during water loss.

7. Heat shock protein 70

Heat shock proteins (HSPs) protect integrity of proteome and help cell survival during stresses. Overexpression of HSP genes can increase the lifespans of *C. elegans* and *D. melanogaster*[33,34]. Two sets of proteins annotated as HSP70 by InterProScan v5.23 and BLASTP separately were retrieved, compared, and merged. The differences between the two sets of HSP70 proteins were manually examined and non-HSP70 proteins were removed. Fifty HSP70 proteins were identified in *A. avenae* (**Supplementary Data 6**). A total of 39 HSP70 sequences of human, yeast, fruit fly and nematodes were collected from HSP70 Sequence Database (http://bioinfo.med.utoronto.ca/HSP70/). Multiple protein sequence alignment was performed by MAFFT v7.046b. Phylogenetic tree was generated by RAxML v 8.1.3

package. Gamma model of rate heterogeneity and DAYHOFF model for amino acid substitution matrix were applied. Then 1,000 rapid bootstrap inferences were executed for large scale maximum likelihood analyses. The phylogenetic tree was displayed by EvolView[28] (**Supplementary Figure 24**).

8. Kinome analysis

Protein phosphorylation plays key roles in signal transduction pathways and cellular processes. The protein kinase domains of protein kinases are relatively conserved during evolution. To identify kinome in *A. avenae*, we aligned the *A. avenae* proteome to multilevel profile HMM library to be classified to each specific protein kinase family[35] by HMMER v3.0 program (E_value < 1e-5). A total of 767 conventional protein kinases (EPKs) and 19 atypical protein kinases (APKs) were grouped in *A. avenae* (**Supplementary Table 10**).

9. Pathways

9.1. Construction of *A. avenae* database for pathway analysis

The C-Elegans5.1 Pathway Studio database from Elsevier contains molecular interactions extracted by natural processing technology for all species from over 120,000 Pubmed abstracts found by keyword search for *Caenorhabditis* OR *elegans* OR *nematode* OR *Worm* and from more than 18,000 full-length open access articles available in Pubmed Central. Proteins in the C-Elegans5.1 database are annotated with Entrez Gene and GenBank identifiers. To facilitate analysis of *A. avenae* genome we added to C-Elegans5.1 database annotated proteins from *B. malayi* and *Caenorhabditis*

briggsae genomes downloaded from NCBI. Proteins orthologs between three nematode species were calculated as described previously[36]. Protein annotation from all orthologs was merged together under one protein identifier in the new database.

The database of *A. avenae* interologs (predicted interactions) and reconstructed pathways were created by adding *A. avenae* identifiers and organism annotation to the orthologous proteins in C-Elegans5.1 database. Prior to ortholog annotation we have clustered splicing variants among predicted 43,192 by comparing *A. avenae* proteome against itself and finding homolog pairs where the shorter protein has over 90% full-length amino acid similarity. This approach identified 29,503 genes which the longest transcript was taken for ortholog calculation.

Orthologs for *A. avenae* proteins in other nematode genomes were calculated using the best reciprocal hit method from full length protein sequence similarities that was calculated from BLAST local alignments as described previously[36]. First, orthologs were calculated between *A. avenae* and *C. elegans* and then additional orthologs were identified in *B. malayi* and *C. briggsae* genomes. The final database contained 54,821 proteins from four nematode genomes where 4,650 proteins annotated with all four organisms, 8,542 proteins annotated with at least three organisms and 8,558 *A. avenae* proteins had orthologs in other genomes.

To identify paralog families in *A. avenae* genome, we used BLASTP program to calculate all protein homologs in *A. avenae* genome and then selected only homologs that have 30% shared amino acid similarity calculated as the average sequence similarity between two homologs. Paralog pairs were imported into *A. avenae* database

as new type of interaction called “Paralog”. Protein functional families were identified as clusters in the global Paralog network using direct force layout algorithm. To assign biological function to each paralog cluster we found Gene Ontology groups enriched by the proteins in the cluster or simply inspected available functional annotation for protein in the cluster.

9.2. Metabolic reconstruction of *A. avenae*

C-Elegans5.1 database from Elsevier contains collection of 298 metabolic pathways manually constructed for different organisms using public data. The pathways and related heatmaps involved in anhydrobiosis were listed in **Supplementary Figures S8-S22**. We have focused on reconstruction of pathways relevant to anhydrobiosis: trehalose metabolism, ROS metabolism, and glutathione metabolism. Each pathway is a collection of ontology classes called Functional Class representing metabolic enzymes connected by corresponding Chemical reactions relations. Every Functional Class in the database can contain unlimited number of protein members performing corresponding enzymatic activity. Usually Functional Class members include paralogs of catalytic, scaffolding and regulatory subunits necessary to perform enzymatic activity. Metabolic reconstruction in Pathway Studio database for *A. avenae* consisted of finding pathways containing Functional Class with protein members from *A. avenae* genome. Pathways containing at least one Functional Class with no *A. avenae* orthologs were manually curated to achieve one of the following three outcomes: a) close the gap by finding members in *A. avenae* genome and adding them to empty functional classes b) dismiss entire pathway if gap cannot be closed c) remove enzymatic step if empty

functional class represents redundant path in the pathway.

9.3. DNA repair pathway reconstruction for *A. avenae*

We have used canonical DNA repair pathways available in Pathway Studio Mammal database to reconstruct DNA repair for *A. avenae*. Homologs for human DNA repair proteins were identified using BLASTP program and physical interactions involved in *A. avenae* DNA repair were predicted as interologs of interactions in human pathway.

Supplementary Figures

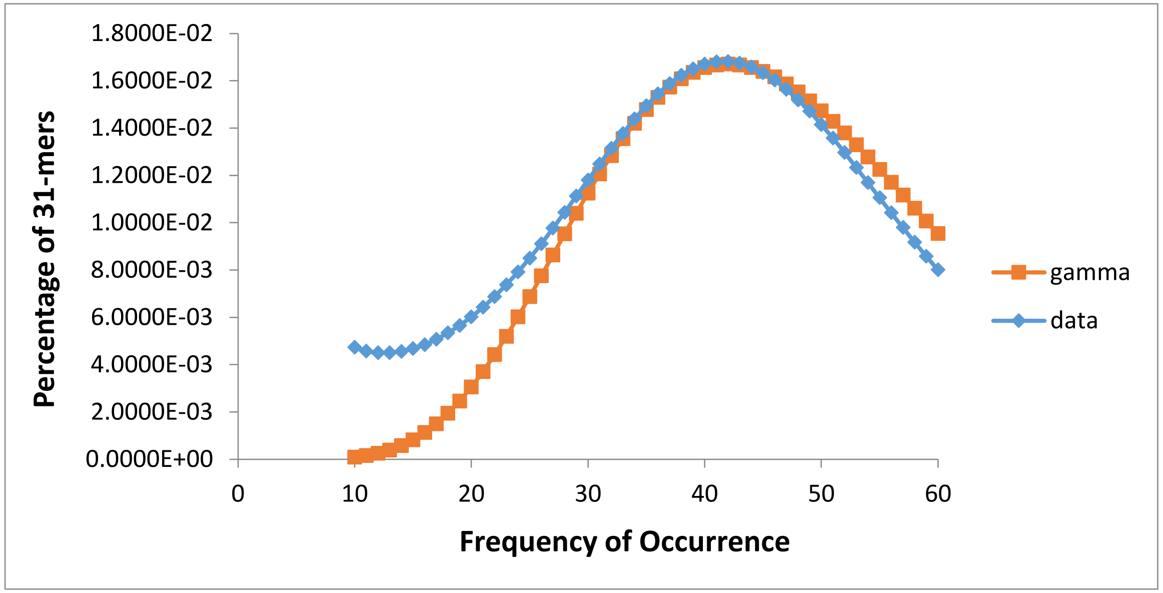

Supplementary Figure 1. Plot of the frequency of multiplicity for 31-mers to estimate *A. avenae* genome size. Distribution of 31-mers of *A. avenae* genome fits gamma distribution. Red square: Gamma distribution fit; Blue diamond: 31-mers.

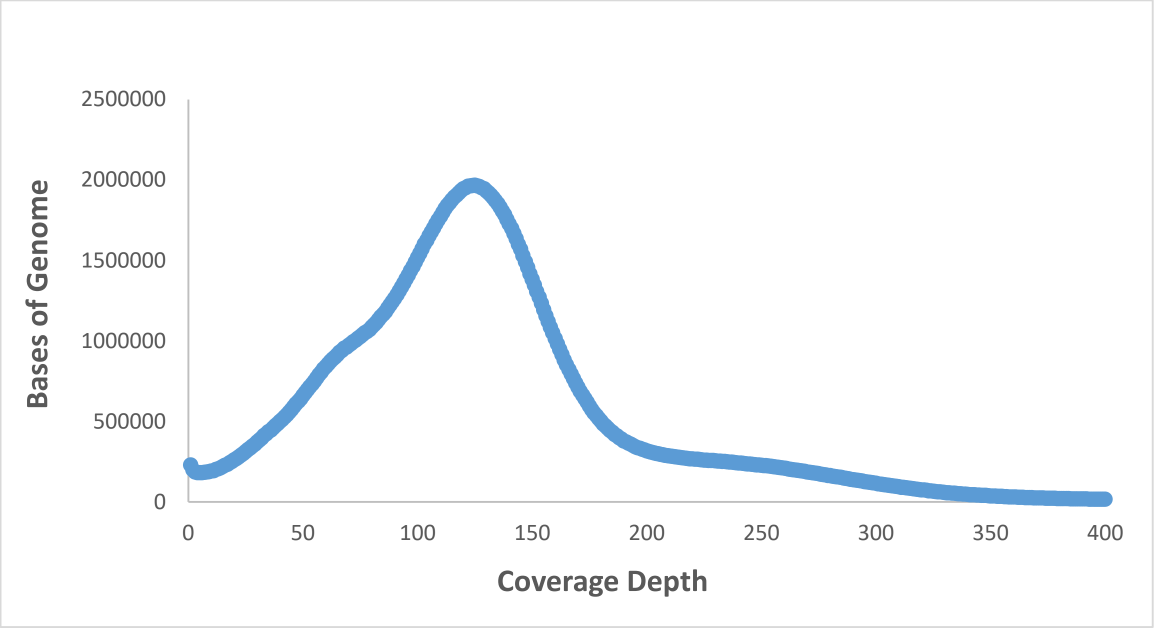

Supplementary Figure 2. The depth of sequencing coverage across the assembled *A. avenae* genome.

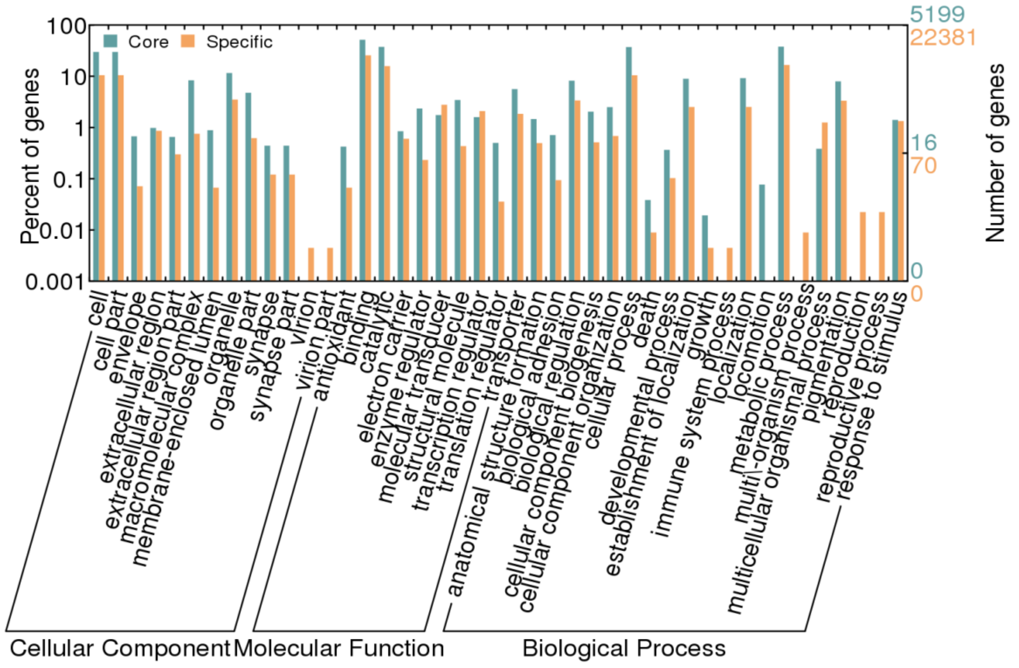

Supplementary Figure 3. Comparison of Gene Ontology enrichments of core orthologs (label: Core, light sea green color) and *A. avenae* species specific genes (label: Specific, orange color).

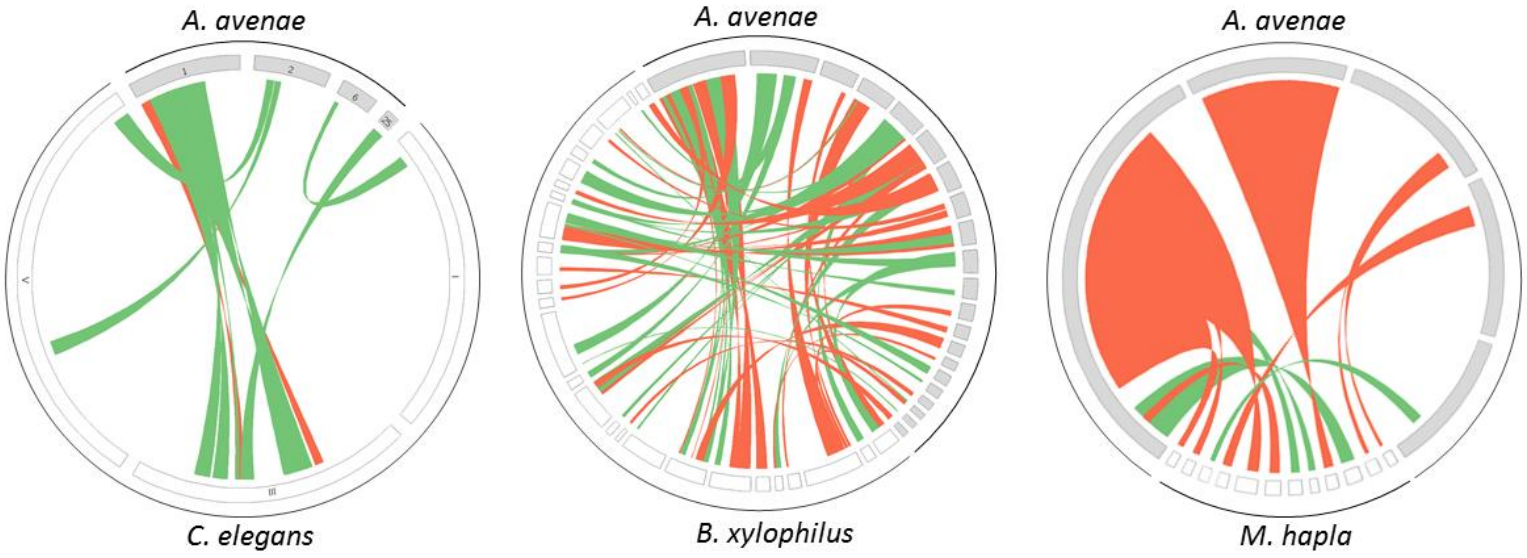

Supplementary Figure 4. Synteny plots between *A. avenae* and *C. elegans*, *B. xylophilus*, or *M. hapla*.

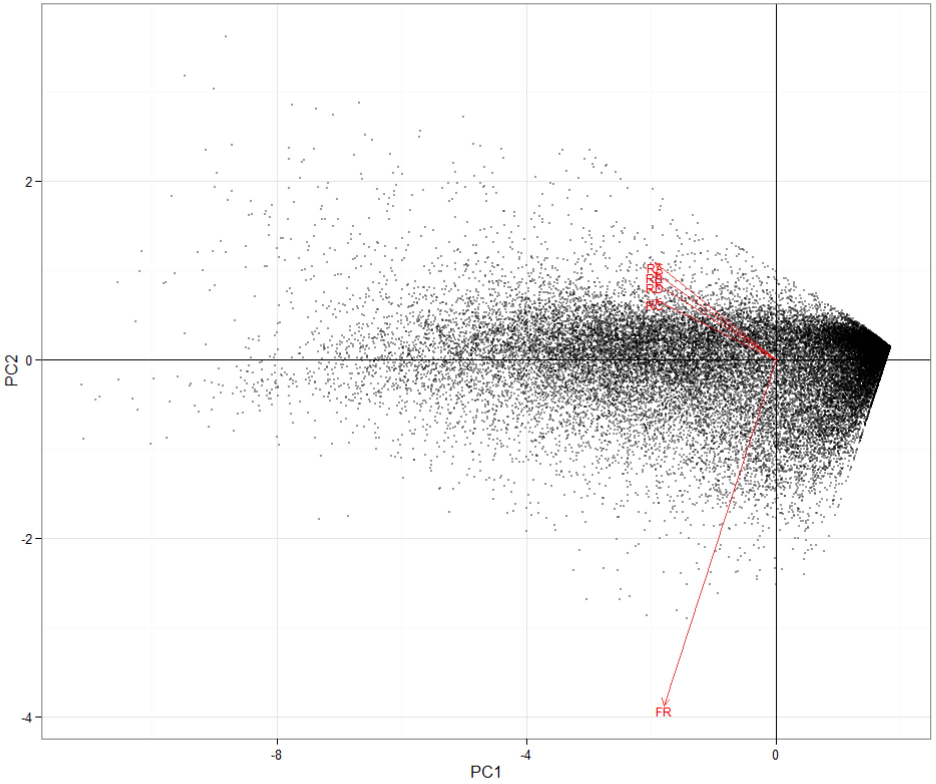

Supplementary Figure 5. Principal component analysis (PCA) plots of log-transformed differential gene expression values exploring the relationships of *A. avenae* gene-level features under the conditions including 100% (FR), 97% (RA), 85% (RB), 40% (RC), and 0% (RD) relative humidity. Arrows represent coordinate axes for each condition. The plot showed that differential expression patterns in dehydrated *A. avenae* are distinct from that in normal *A. avenae*.

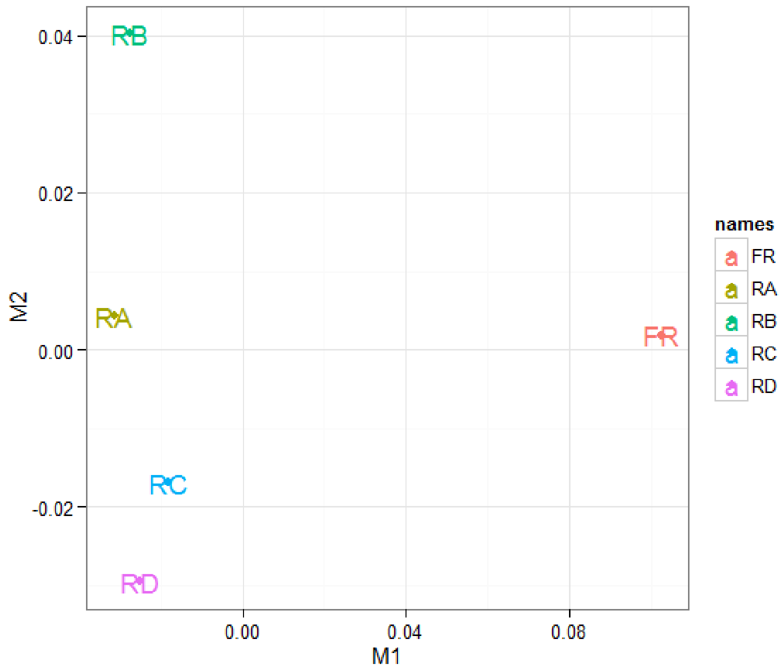

Supplementary Figure 6. Multi-dimensional scaling (MDS) plots of log-transformed differential gene expression (DGE) values exploring the relationships of *A. avenae* gene-level features under the conditions including 100% (FR), 97% (RA), 85% (RB), 40% (RC), and 0% (RD) relative humidity.

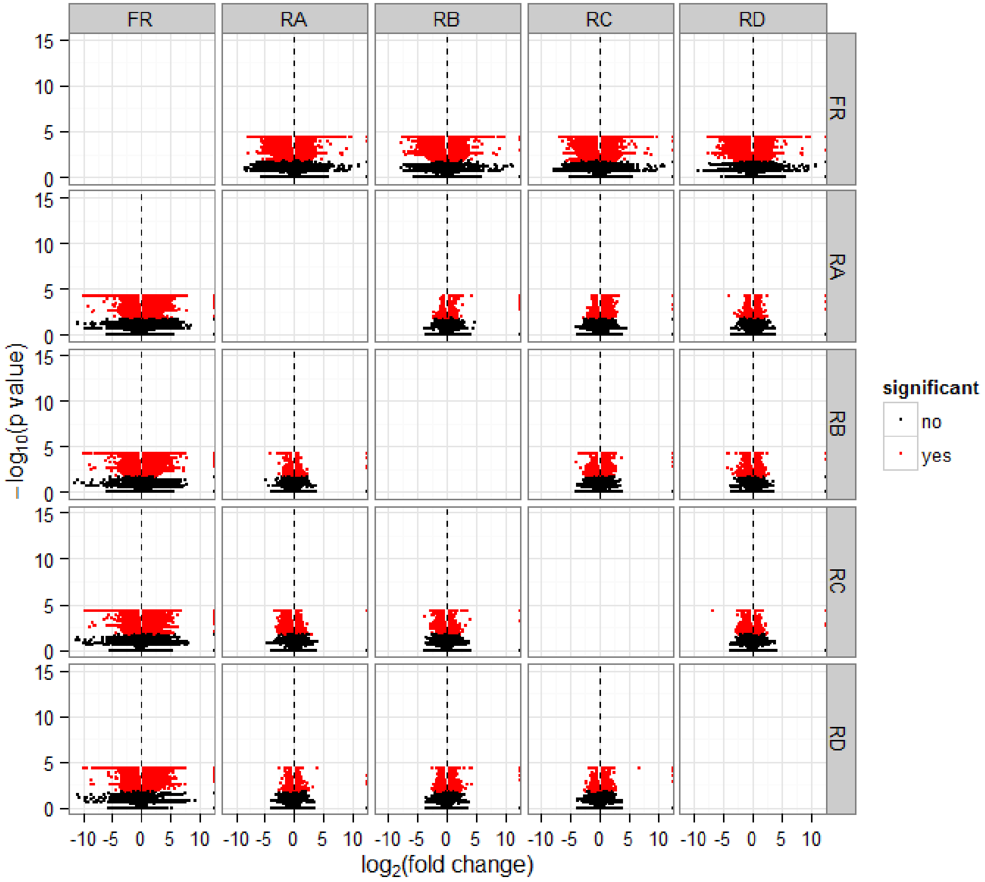

Supplementary Figure 7. Volcano plots of log_2 gene expression fold-change (x-axis) versus $–log_{10}$ p value (y-axis) in RNA-seq analysis of *A. avenae* under various conditions including 100% (FR), 97% (RA), 85% (RB), 40% (RC), and 0% (RD) relative humidity. Significance of differential gene expression was defined by setting alpha to 0.05 (n=3 biologically independent samples).

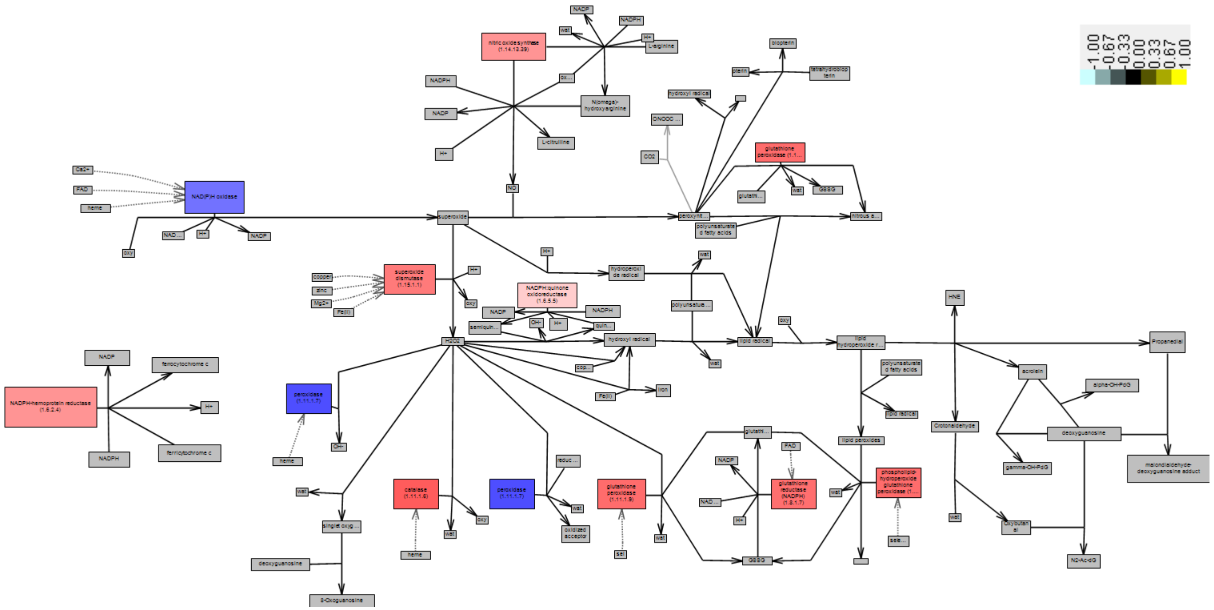

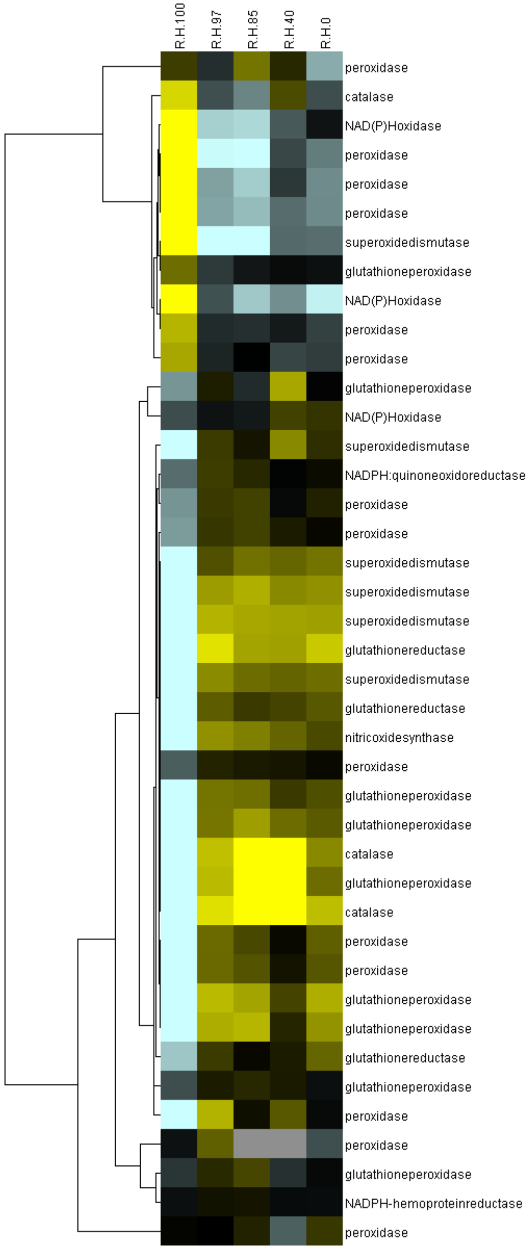

Supplementary Figure 8. Reconstruction of reactive oxygen species (ROS) pathway reveals up-regulated **(**red**)** and down-regulated (blue) genes during water loss. Heatmap of differentially expressed genes involved in ROS pathway. The color represents the z-scores of the expression values of the genes, which were calculated as the mean-centered FPKM values.

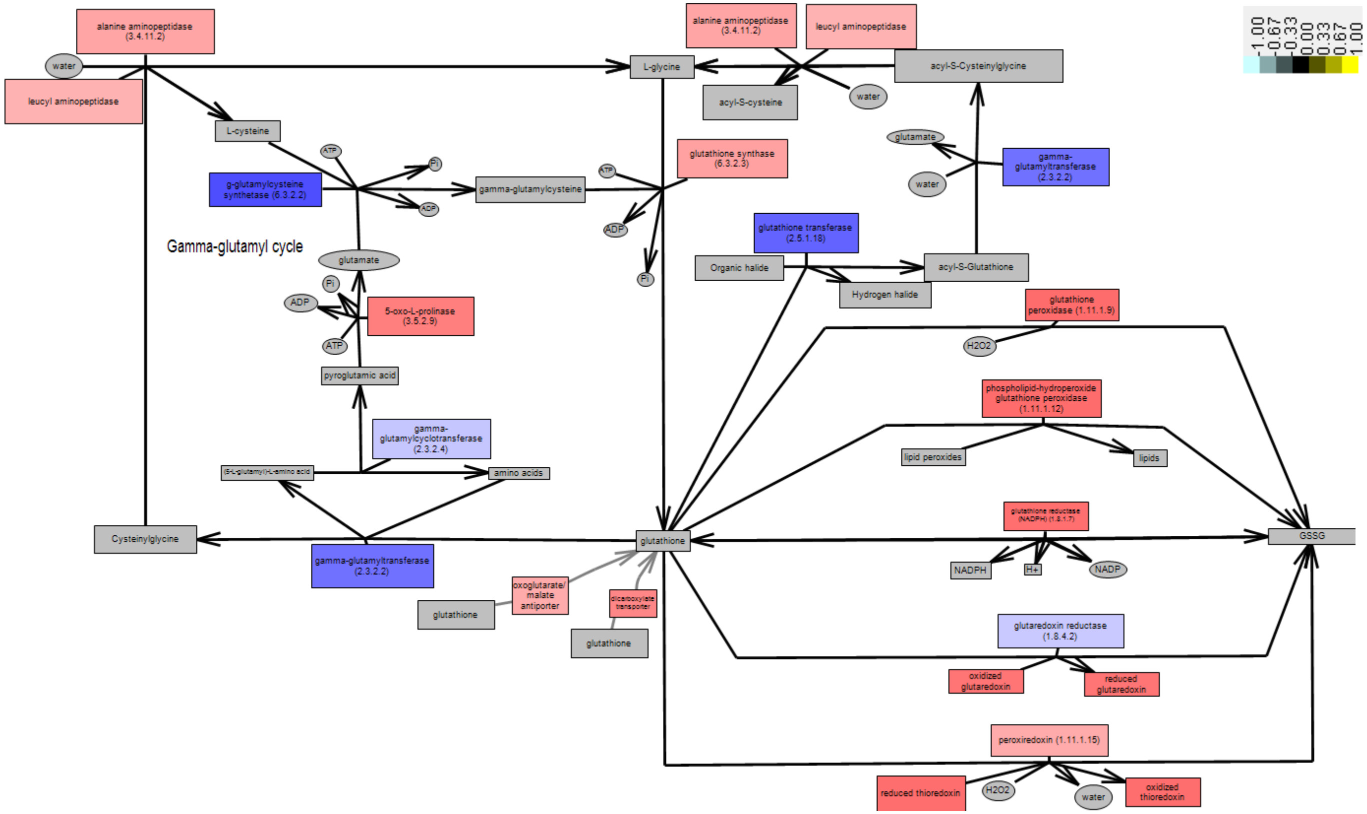

Gamma-glutamyl cycle
alanine aminopeptidase (3.4.11.2)
leucyl aminopeptidase
water
L-glycine
L-cysteine
g-glutamylcysteine synthetase (6.3.2.2)
gamma-glutamylcysteine
glutathione synthase (6.3.2.3)
acyl-S-cysteine
acyl-S-Cysteinylglycine
glutamate
water
gamma-glutamyltransferase (2.3.2.2)
glutathione transferase (2.5.1.18)
Organic halide
acyl-S-Glutathione
Hydrogen halide
glutathione peroxidase (1.11.1.9)
H2O2
5-oxo-L-prolinase (3.5.2.9)
pyroglutamic acid
phospholipid-hydroperoxide glutathione peroxidase (1.11.1.12)
lipid peroxides
lipids
gamma-glutamylcyclotransferase (2.3.2.4)
amino acids
Cysteinylglycine
gamma-glutamyltransferase (2.3.2.2)
glutathione
GSSG
oxoglutarate/ malate antiporter
glutathione reductase (NADPH) (1.8.1.7)
NADPH
H+
NADP
glutaredoxin reductase (1.8.4.2)
oxidized glutaredoxin
reduced glutaredoxin
peroxiredoxin (1.11.1.15)
reduced thioredoxin
H2O2
water
oxidized thioredoxin
ATP
ADP
Pi

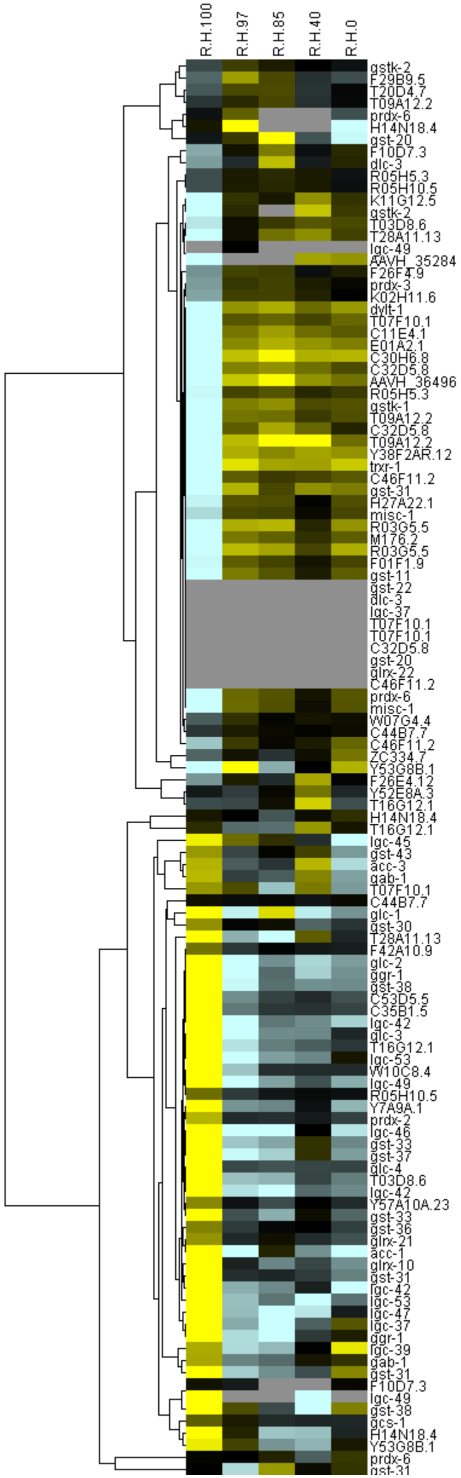

R.H.100
R.H.97
R.H.85
R.H.40
R.H.0
-1.00
-0.67
-0.33
0.00
0.33
0.67
1.00

Supplementary Figure 9. Reconstruction of glutathione metabolism pathway reveals up-regulated **(**red**)** and down-regulated (blue) genes during water loss. Heatmap of differentially expressed genes involved in glutathione metabolism pathway. The color represents the z-scores of the expression values of the genes, which were calculated as the mean-centered FPKM values.

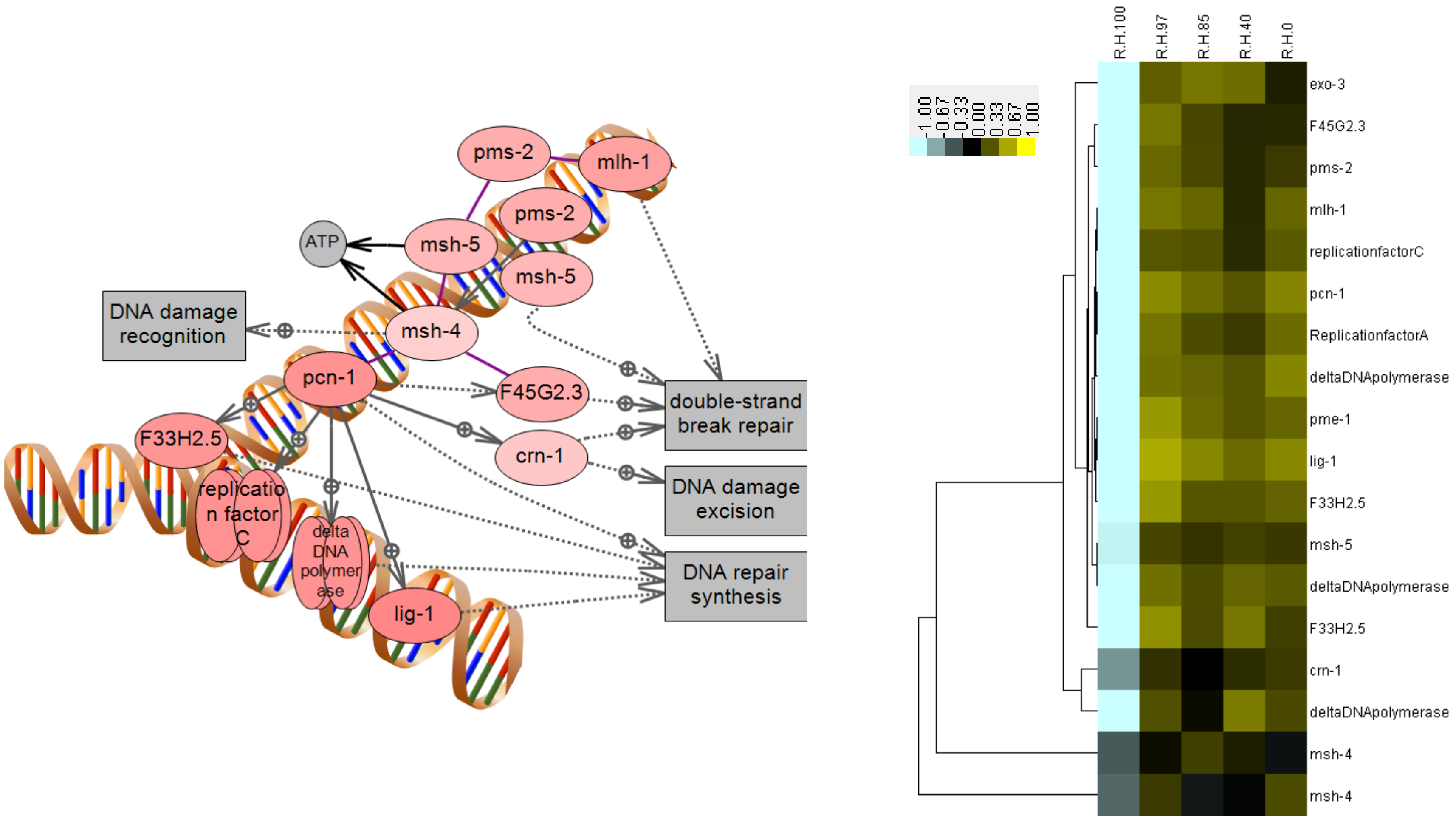

Supplementary Figure 10. Reconstruction of DNA single-strand mismatch pathway reveals up-regulated (red) genes during water loss. Heatmap of differentially expressed genes involved in DNA single-strand mismatch pathway. The color represents the z-scores of the expression values of the genes, which were calculated as the mean-centered FPKM values.

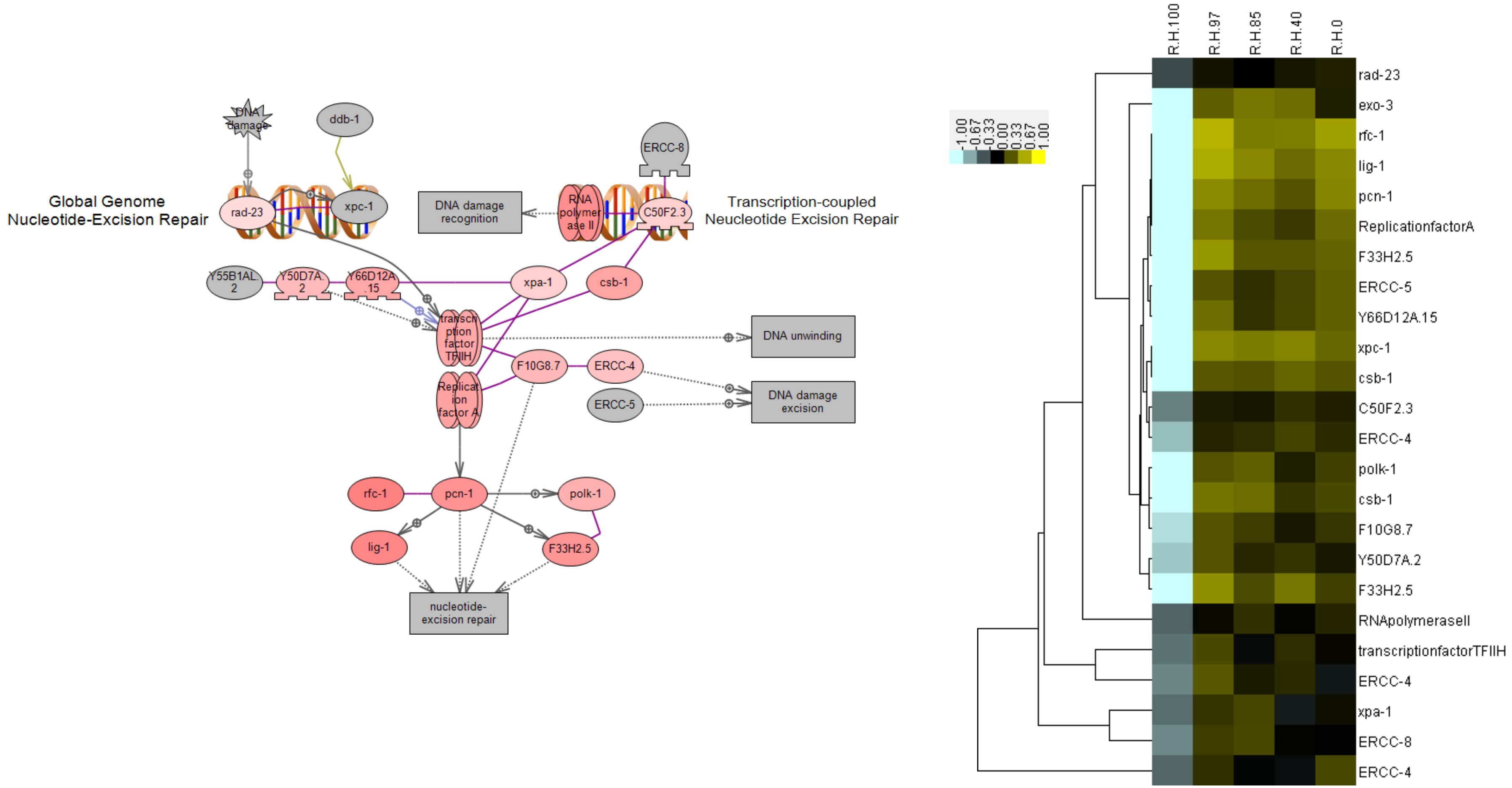

Supplementary Figure 11. Reconstruction of single-strand nucleotide excision DNA repair pathway reveals up-regulated (red) genes during water loss. Heatmap of differentially expressed genes involved in single-strand nucleotide excision DNA repair pathway. The color represents the z-scores of the expression values of the genes, which were calculated as the mean-centered FPKM values.

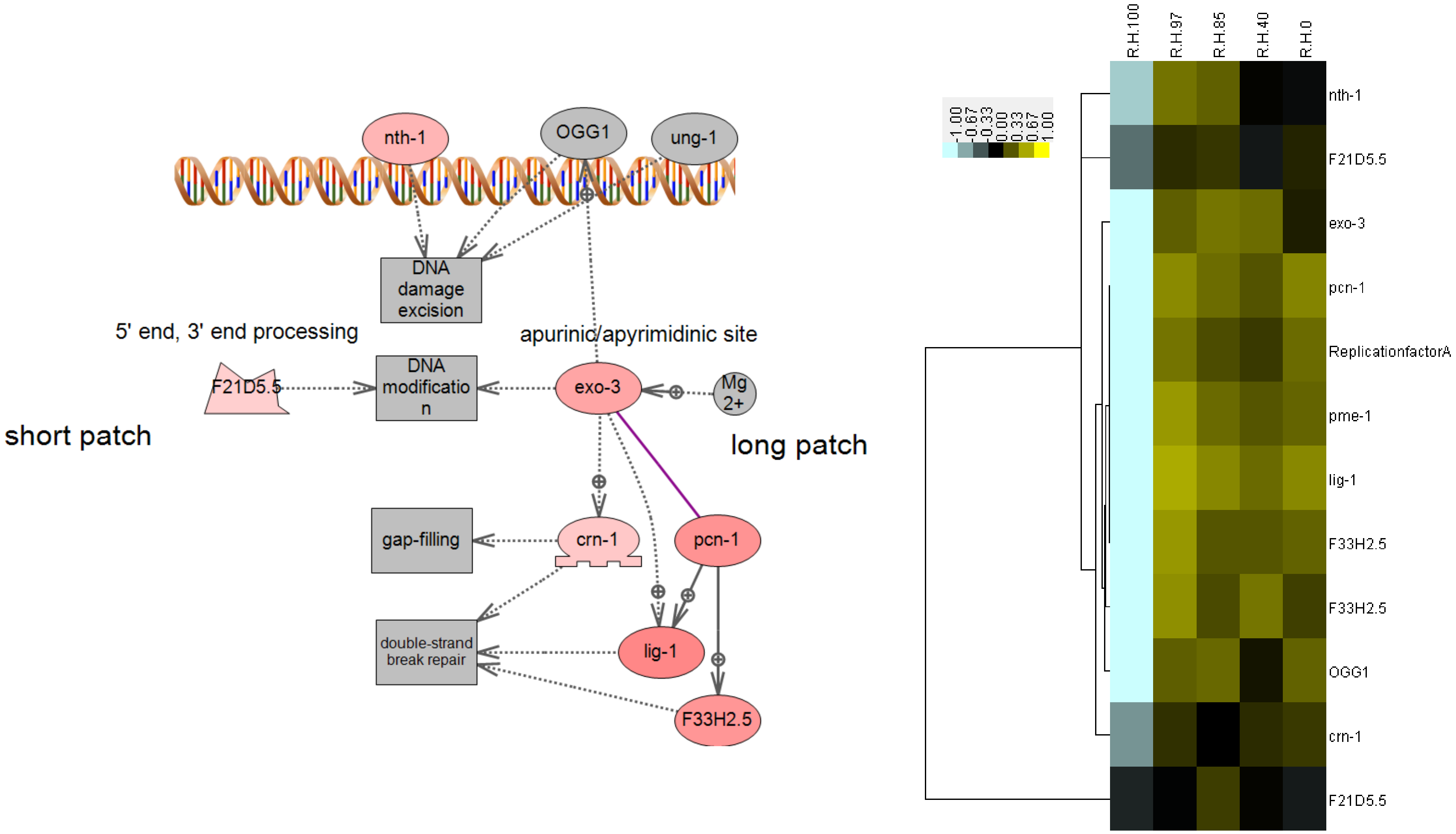

Supplementary Figure 12. Reconstruction of single-strand base excision DNA repair pathway reveals up-regulated (red) genes during water loss. Heatmap of differentially expressed genes involved in single-strand base excision DNA repair pathway. The color represents the z-scores of the expression values of the genes, which were calculated as the mean-centered FPKM values.

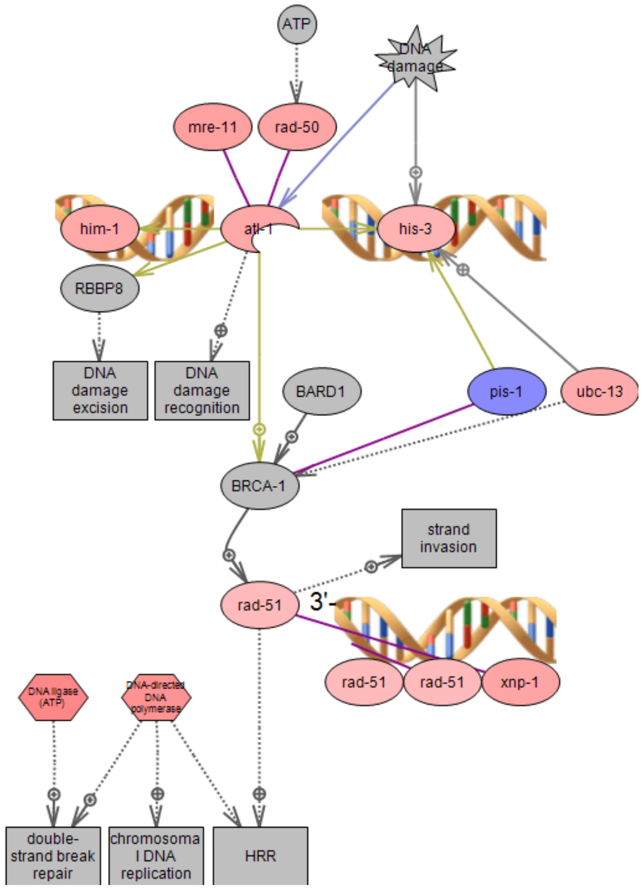

ATP
DNA damage
mre-11
rad-50
him-1
atl-1
his-3
RBBP8
DNA damage excision
DNA damage recognition
BARD1
pis-1
ubc-13
BRCA-1
strand invasion
rad-51
3'
rad-51
rad-51
xnp-1
DNA ligase (ATP)
DNA-directed DNA polymerase
double-strand break repair
chromosomal DNA replication
HRR

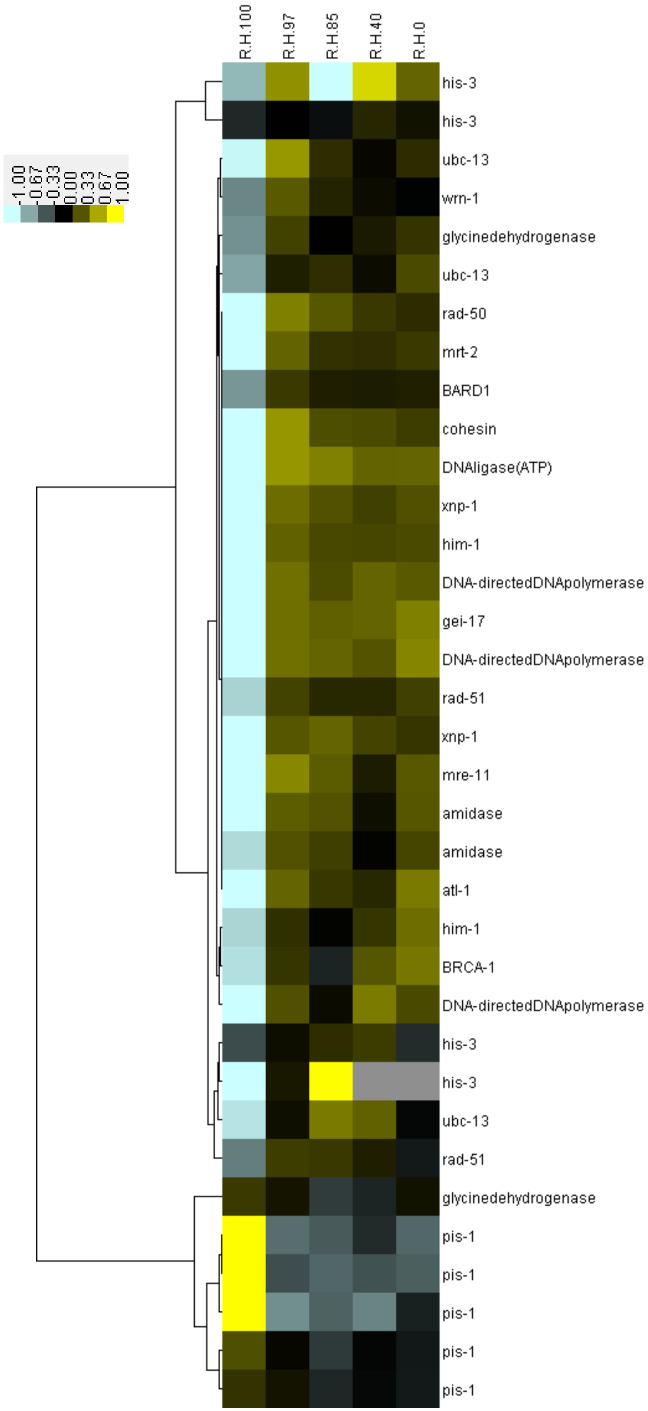

-1.00
-0.67
-0.33
0.00
0.33
0.67
1.00
R.H.100
R.H.97
R.H.85
R.H.40
R.H.0
his-3
his-3
ubc-13
wrn-1
glycinedehydrogenase
ubc-13
rad-50
mrt-2
BARD1
cohesin
DNAligase(ATP)
xnp-1
him-1
DNA-directedDNApolymerase
gei-17
DNA-directedDNApolymerase
rad-51
xnp-1
mre-11
amidase
amidase
atl-1
him-1
BRCA-1
DNA-directedDNApolymerase
his-3
his-3
ubc-13
rad-51
glycinedehydrogenase
pis-1
pis-1
pis-1
pis-1
pis-1

Supplementary Figure 13. Reconstruction of double-strand DNA homologous repair pathway reveals up-regulated **(**red**)** and down-regulated (blue) genes during water loss. Heatmap of differentially expressed genes involved in double strand DNA homologous repair pathway. The color represents the z-scores of the expression values of the genes, which were calculated as the mean-centered FPKM values.

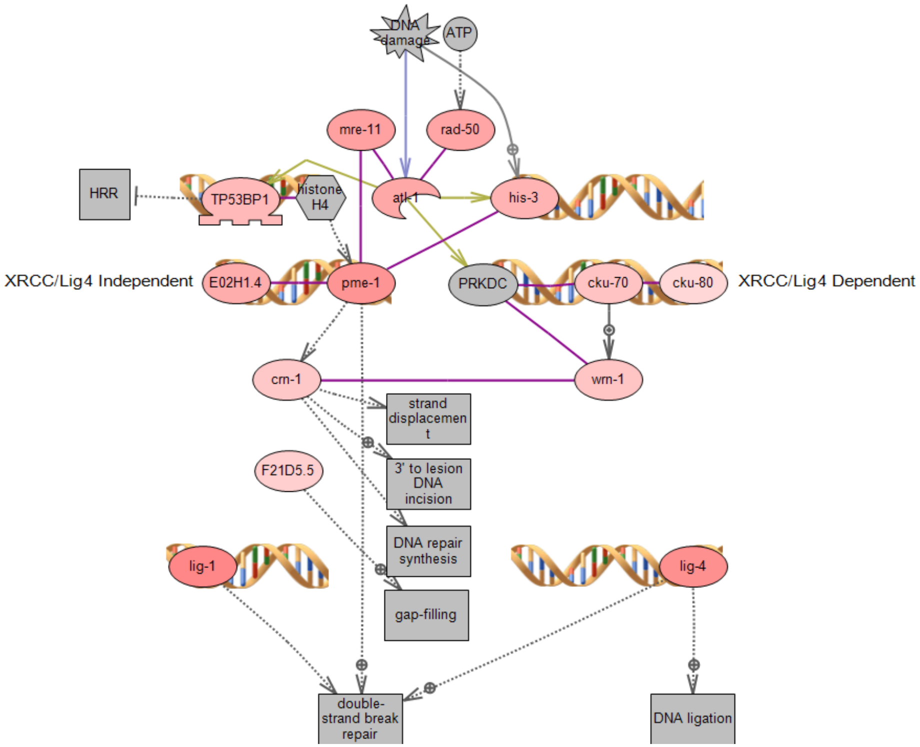
DNA damage
ATP
mre-11
rad-50
HRR
TP53BP1
histone H4
atl-1
his-3
XRCC/Lig4 Independent
E02H1.4
pme-1
PRKDC
cku-70
cku-80
XRCC/Lig4 Dependent
crn-1
wrn-1
strand displacement
F21D5.5
3' to lesion DNA incision
DNA repair synthesis
lig-1
lig-4
gap-filling
double-strand break repair
DNA ligation

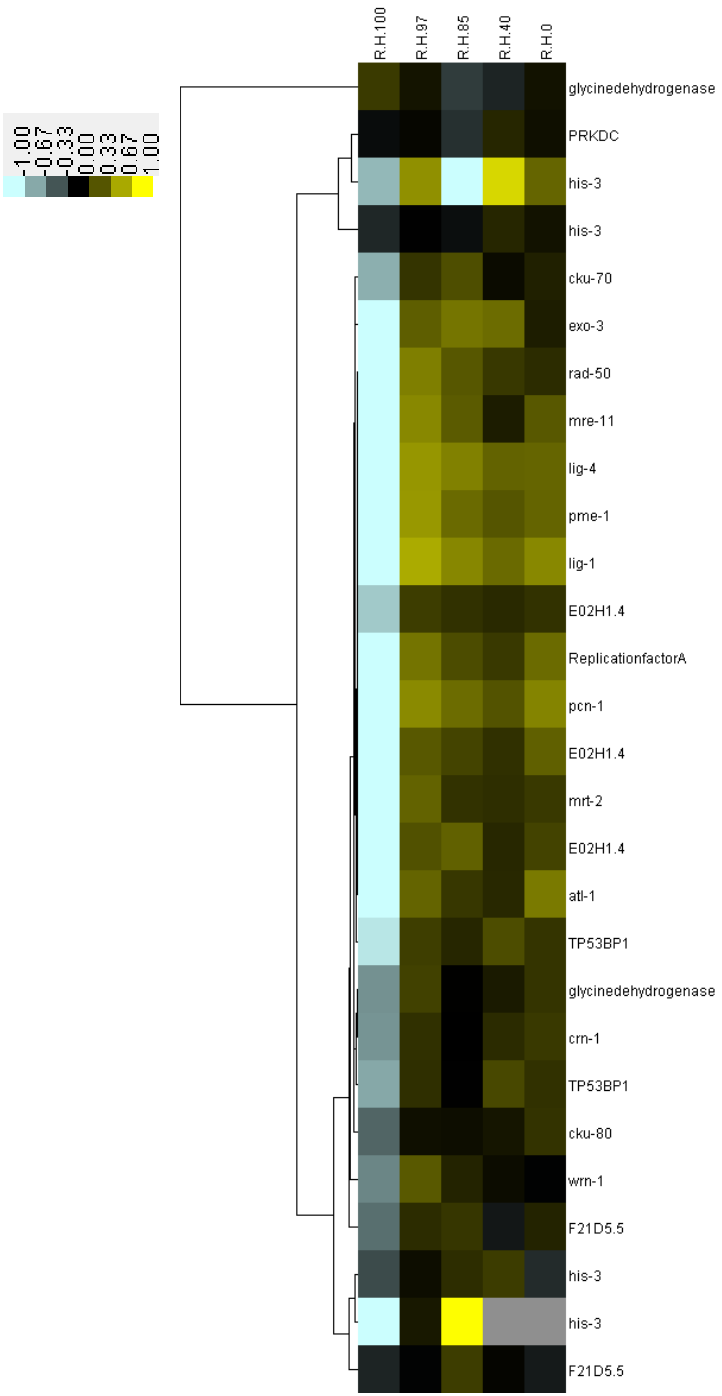
-1.00
-0.67
-0.33
0.00
0.33
0.67
1.00
R.H.100
R.H.97
R.H.85
R.H.40
R.H.0
glycinedehydrogenase
PRKDC
his-3
his-3
cku-70
exo-3
rad-50
mre-11
lig-4
pme-1
lig-1
E02H1.4
ReplicationfactorA
pcn-1
E02H1.4
mrt-2
E02H1.4
atl-1
TP53BP1
glycinedehydrogenase
crn-1
TP53BP1
cku-80
wrn-1
F21D5.5
his-3
his-3
F21D5.5

Supplementary Figure 14. Reconstruction of double-strand DNA non-homologous repair pathway reveals up-regulated **(**red**)** and down-regulated (blue) genes during water loss. Heatmap of differentially expressed genes involved in double-strand DNA non-homologous repair pathway. The color represents the z-scores of the expression values of the genes, which were calculated as the mean-centered FPKM values.

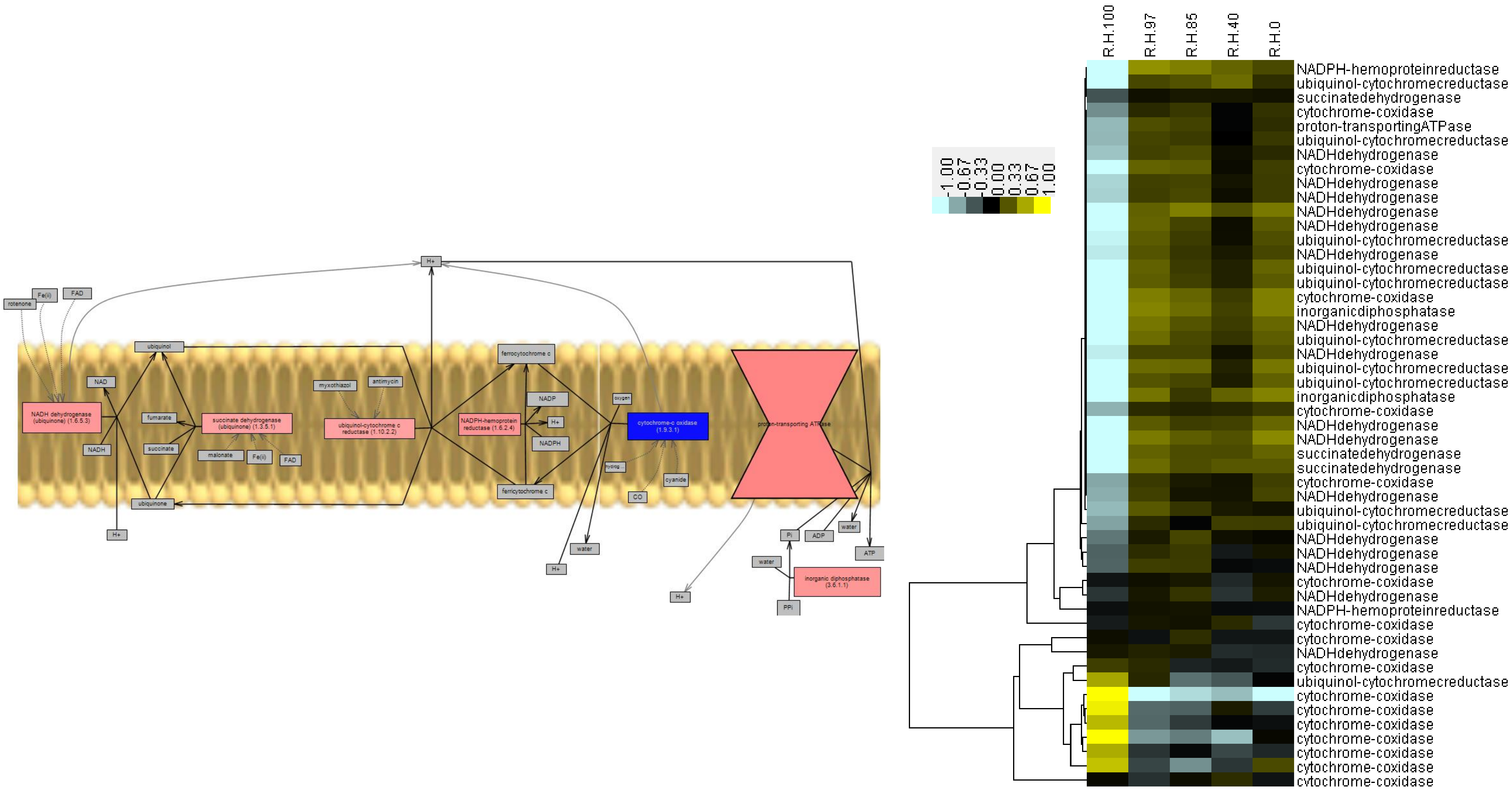

Supplementary Figure 15. Reconstruction of electron transport chain pathway reveals up-regulated **(**red) and down-regulated (blue) genes during water loss. Heatmap of differentially expressed genes involved in electron transport chain pathway. The color represents the z-scores of the expression values of the genes, which were calculated as the mean-centered FPKM values.

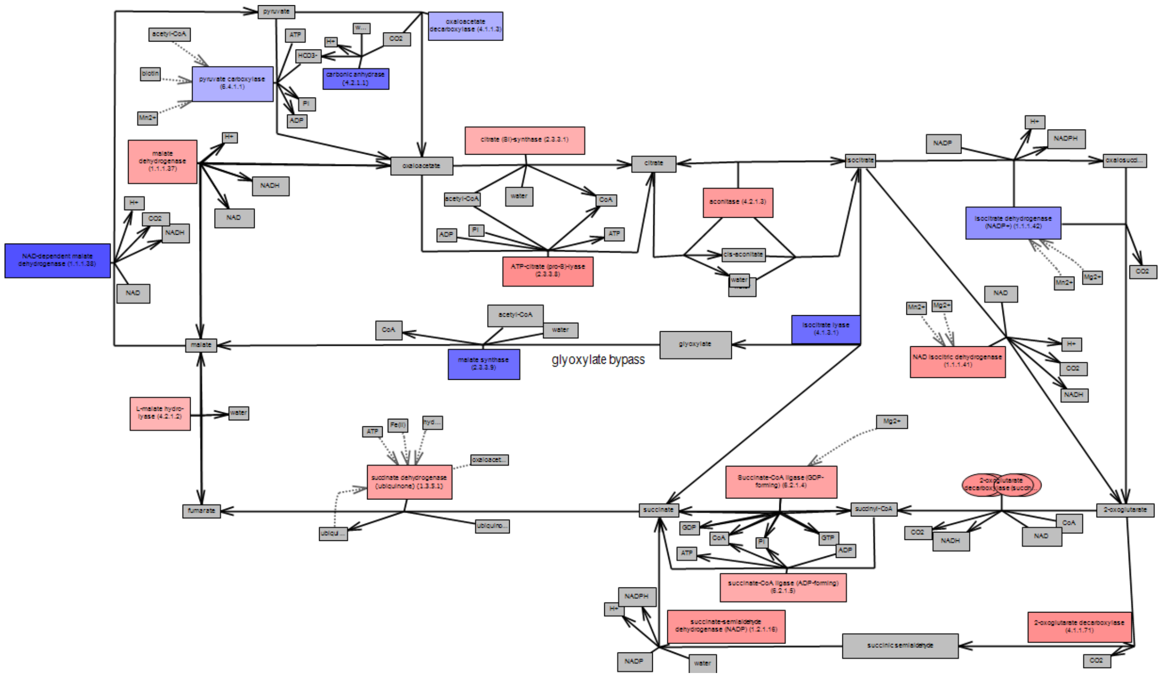

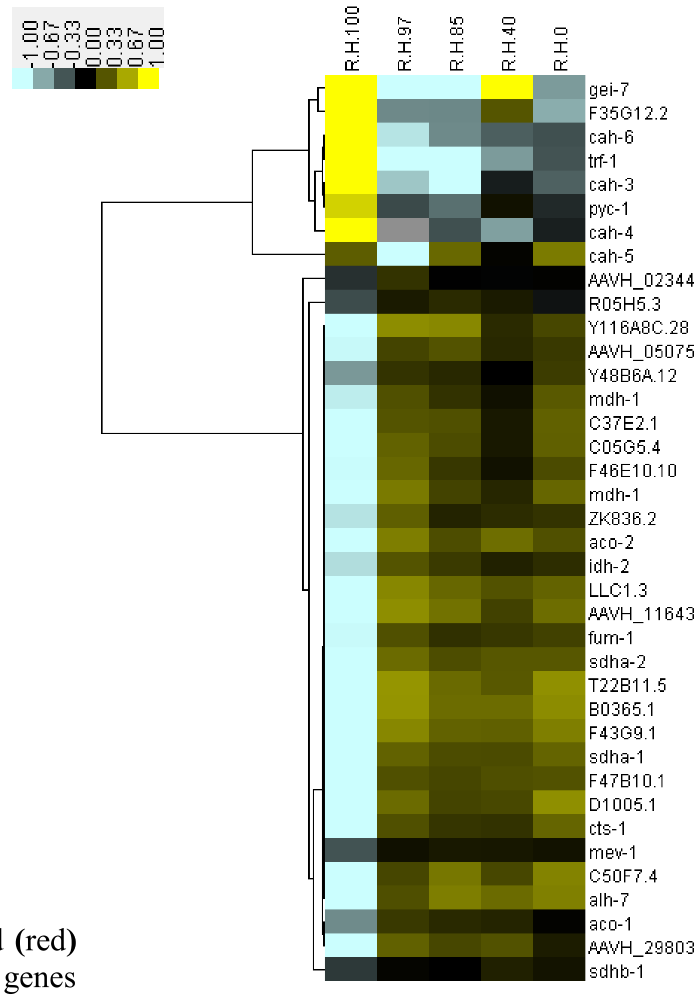

Supplementary Figure 16. Reconstruction of TCA cycle pathway reveals up-regulated (red) and down-regulated (blue) genes during water loss. Heatmap of differentially expressed genes involved in TCA cycle pathway. The color represents the z-scores of the expression values of the genes, which were calculated as the mean-centered FPKM values.

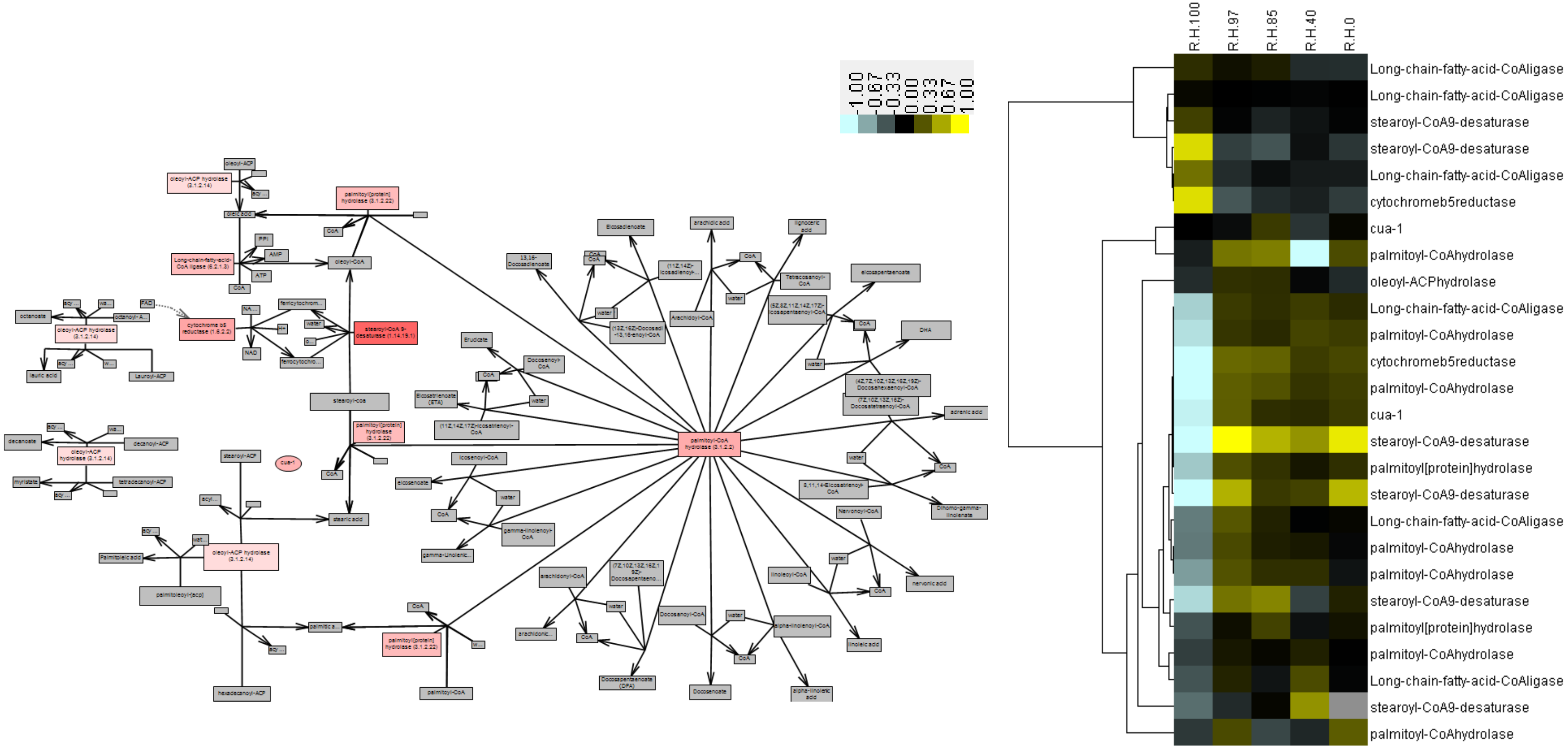

Supplementary Figure 17. Reconstruction of fatty acid synthesis pathway reveals up-regulated **(red)** genes during water loss. Heatmap of differentially expressed genes involved in fatty acid synthesis pathway. The color represents the z-scores of the expression values of the genes, which were calculated as the mean-centered FPKM values.

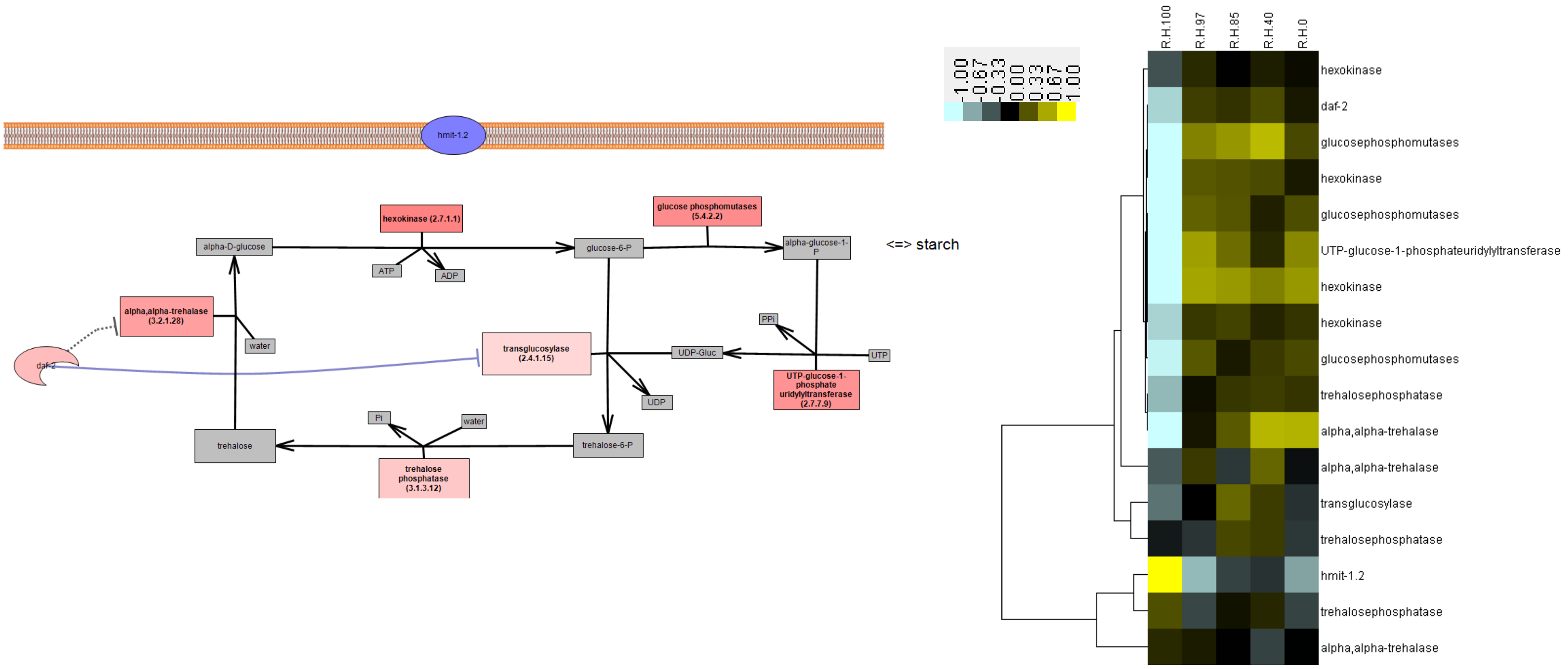

Supplementary Figure 18. Reconstruction of trehalose metabolism pathway reveals up-regulated **(**red) and down-regulated (blue) genes during water loss. Heatmap of differentially expressed genes involved in trehalose metabolism pathway. The color represents the z-scores of the expression values of the genes, which were calculated as the mean-centered FPKM values.

Supplementary Figure 19. Heatmap of differentially expressed aquaporin genes. The color represents the z-scores of the expression values of the genes, which were calculated as the mean-centered FPKM values.

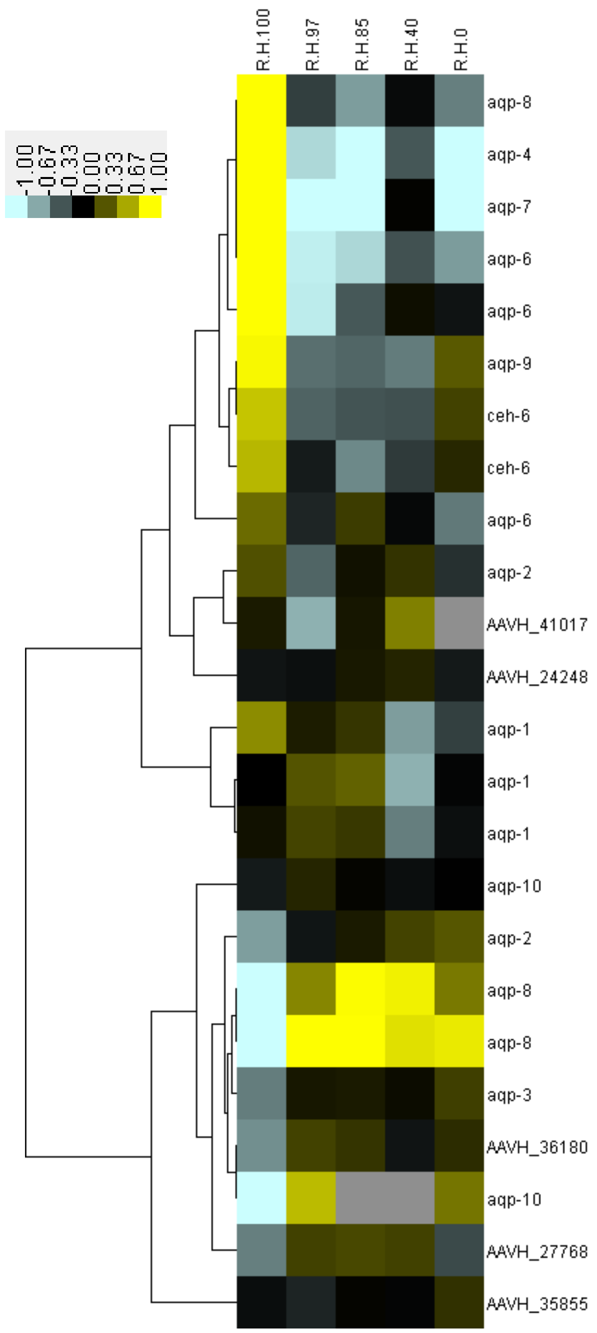

Supplementary Figure 20. Heatmap of differentially expressed LIM genes. The color represents the z-scores of the expression values of the genes, which were calculated as the mean-centered FPKM values.

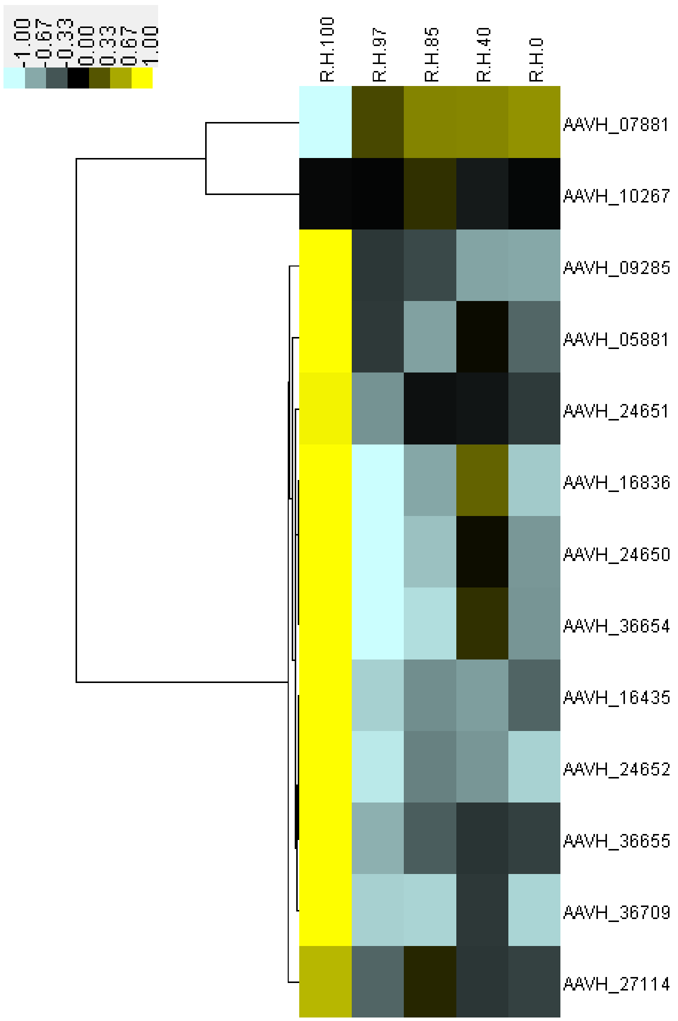

Supplementary Figure 21. Heatmap of differentially expressed DNA topoisomerase genes. The color represents the z-scores of the expression values of the genes, which were calculated as the mean-centered FPKM values.

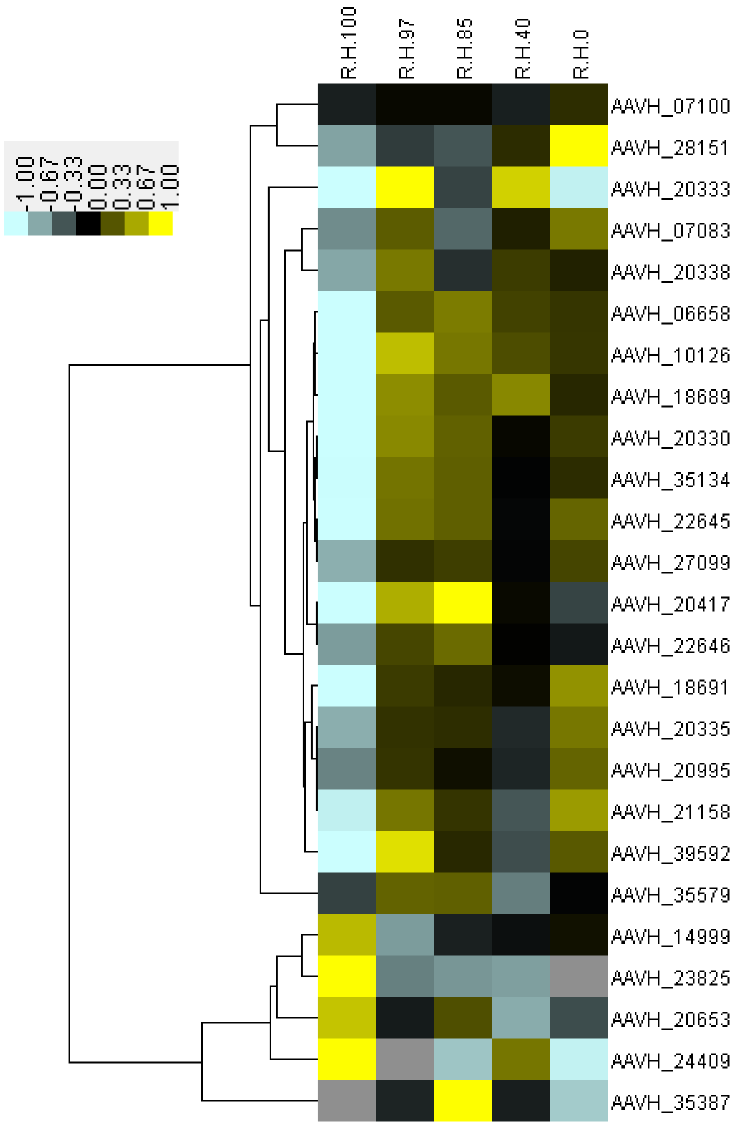

Supplementary Figure 22. Heatmap of differentially expressed LEA genes. The color represents the z-scores of the expression values of the genes, which were calculated as the mean-centered FPKM values.

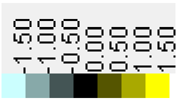

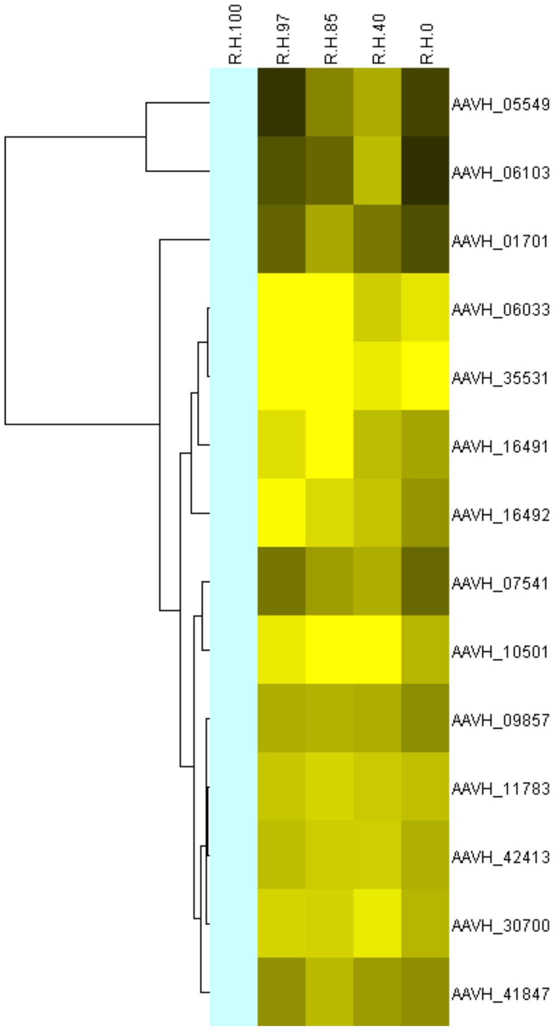

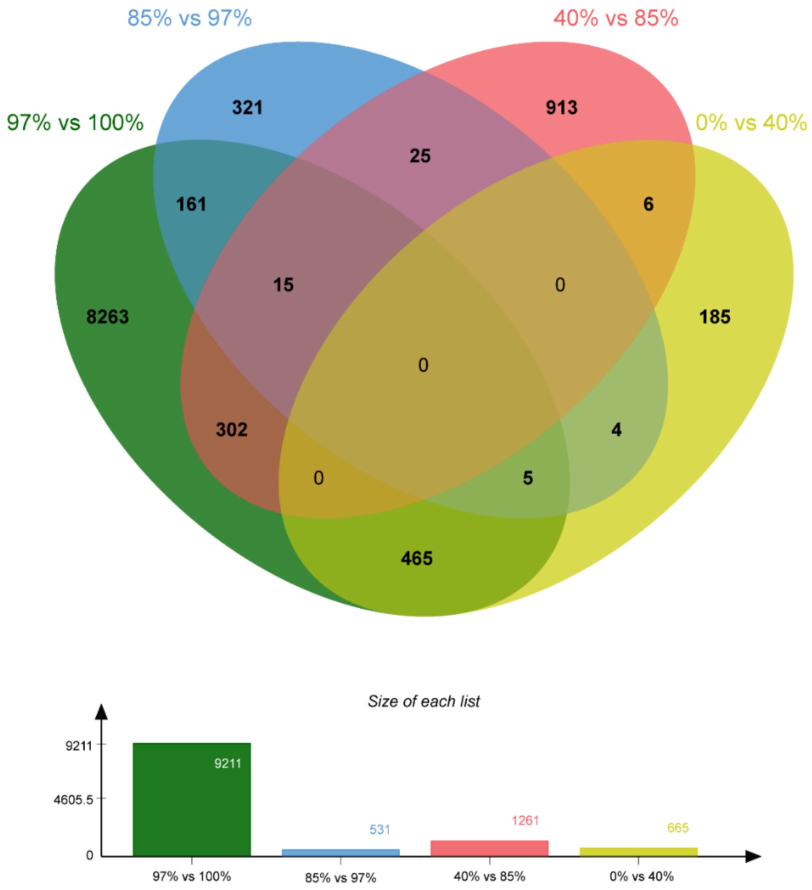

Supplementary Figure 23. Venn diagram showing the numbers of up-regulated genes from *A. avenae* between the different drying regimes.

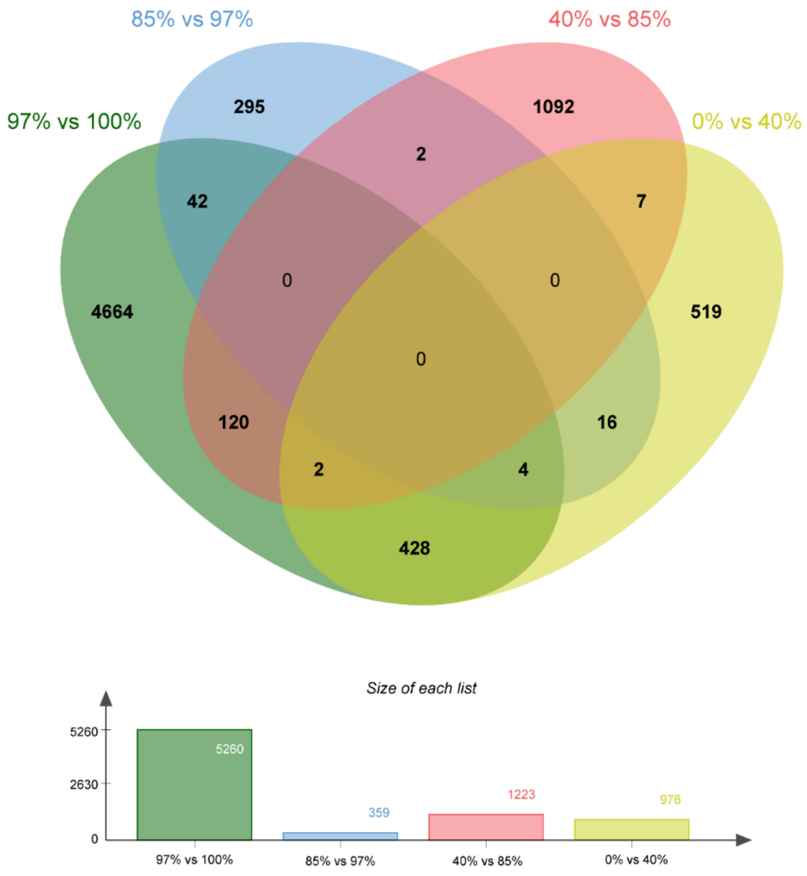

Supplementary Figure 24. Venn diagram showing the numbers of down-regulated genes from *A. avenae* between the different drying regimes.

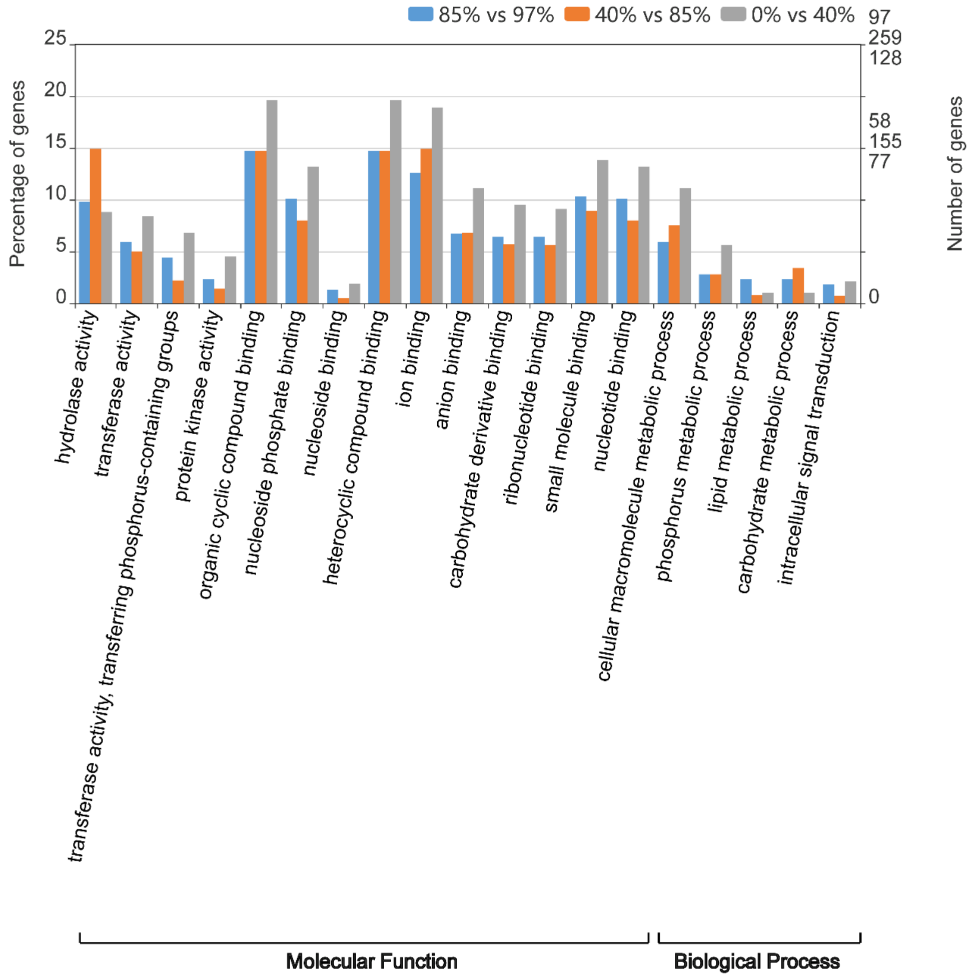

Supplementary Figure 25. Gene Ontology enrichments of up-regulated genes from *A. avenae* between the different drying regimes.

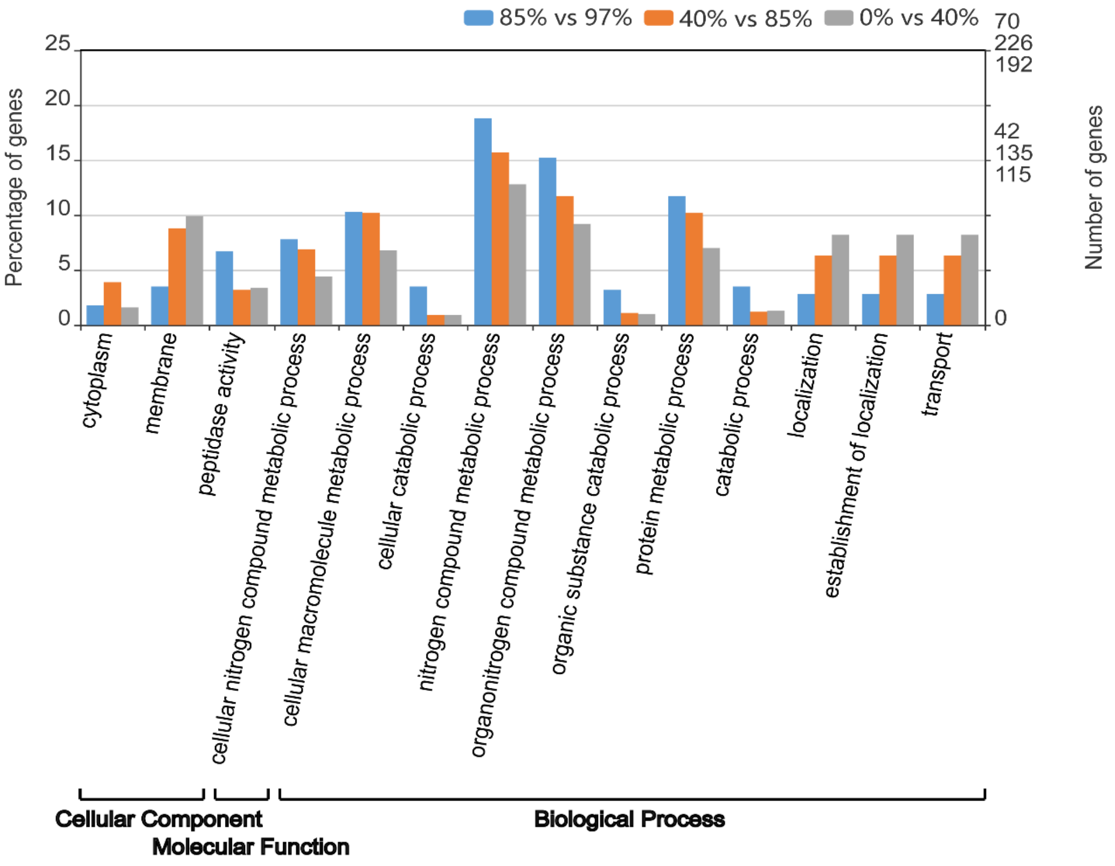

Supplementary Figure 26. Gene Ontology enrichments of down-regulated genes from *A. avenae* between the different drying regimes.

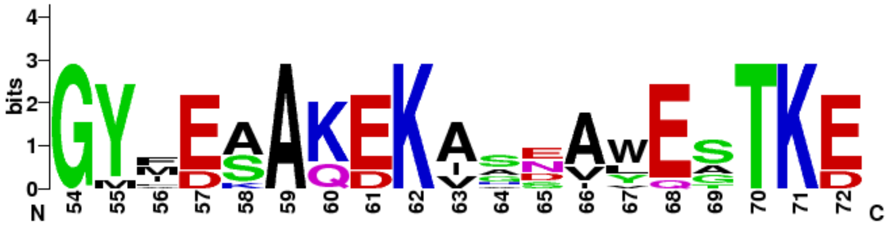

Supplementary Figure 27. Sequence logo pattern of the learned LEA motif profile.

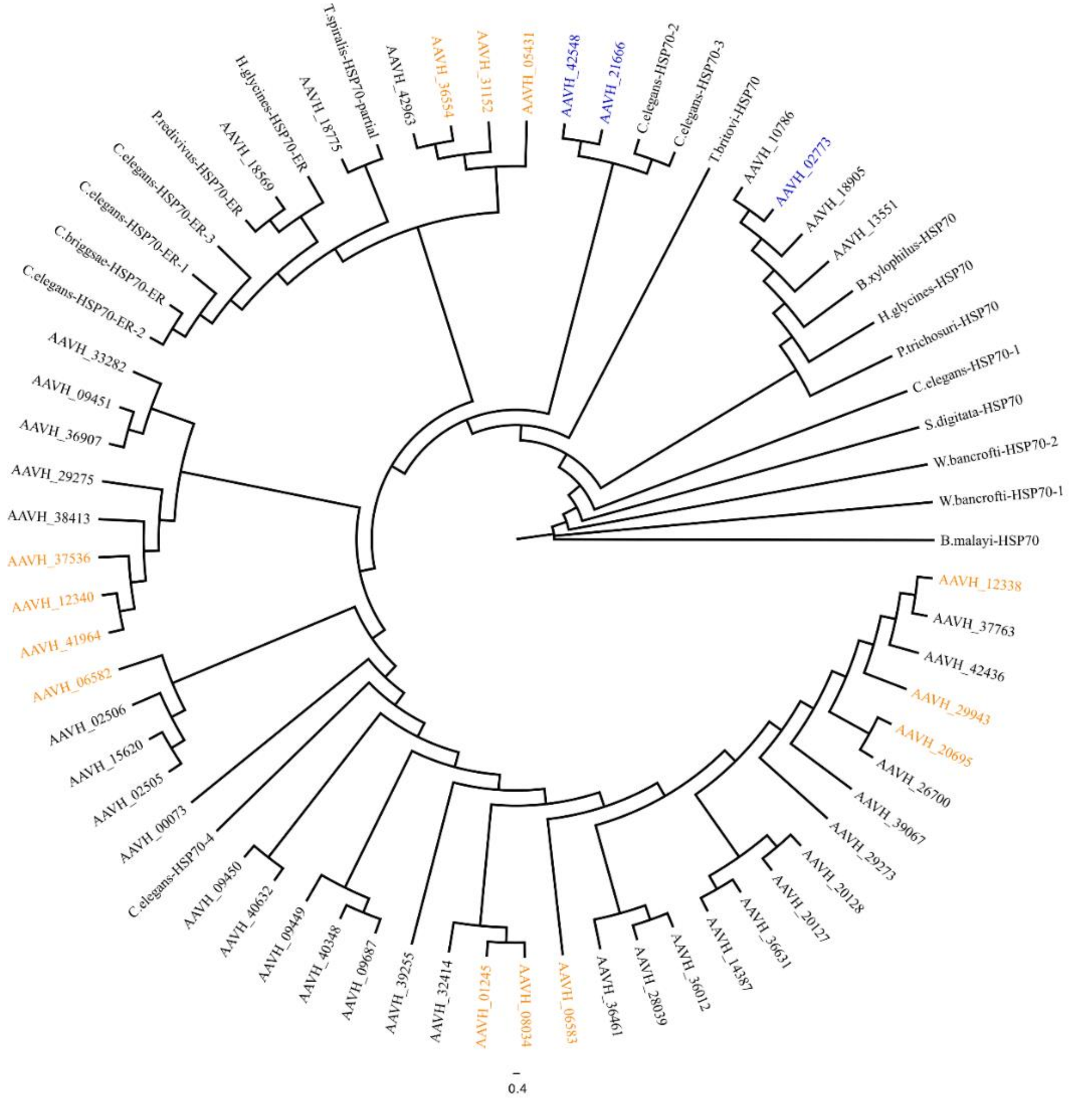

Supplementary Figure 28. Maximum-likelihood phylogenetic tree of HSP70 protein sequences from *A. avenae*, *B. malayi*, *B. xylophilus*, *C. briggsae*, *C. elegans*, *H. glycines*, *P. redivivus*, *P. trichosuri*, *S. digitata*, *T. britovi*, *T. spiralis*, and *W. bancrofti*. During water loss process, up-regulated and down-regulated *A. avenae* HSP70 genes are labeled orange and blue separately.

Supplementary Tables

Supplementary Table 1. Summary of genome sequencing reads and coverage.

Assembler	Library	Number of reads	Number of bases	Coverage
Newbler 2.8	454 shotgun libraries	4299229	1768912454	6.9
	454 8 kb paired-end libraries	2569299	846514699	3.3
	454 20 kb paired-end libraries	3060780	1108576497	4.3
	pseudo read library	1491907	531867167	2.1
Soapdenovo	Illumina 500 bp paired-end library	554236438	~ 41567732850	163

Supplementary Table 2. Properties of the *A. avenae* genome.

Features	*A. avenae*
Assembly	
Estimated size of genome (Mb)	255
Total size of assembled sequence (Mb)	264.8
coverage	~ 180 X
Number of scaffolds	18660
N50 scaffold length (bp)	141564
Maximum length scaffold (Mb)	5.5
Number of bp assembled into scaffolds (Mb)	223.4
Number of bp in gaps (Mb)	31.6
Protein-coding regions	
Number of protein-coding gene models	43192
Protein-coding sequence (% of genome)	15
Gene density (genes per Mb)	169
Max/average protein length (amino acids)	6828/295.5
Number of exons	208243
Mean exon size (bp)	184
Mean number of exons per gene	4.8
Number of bp included in exons	38300385
Number of introns	165051
Mean intron size (bp)	218
Number of bp included in introns	36028257
Mean length of intergenic region (bp)	2038
Operon number	4453

Percent of genes present in operon	23.3
Overall G + C content (%)	49.7
Exons, G + C content (%)	56
Introns, G + C content (%)	44.7
Intergenic regions, G + C content (%)	48.8
Non-protein coding genes	
Transfer RNA (tRNA) genes (+ tRNA pseudogenes)	~492 (+50)
Selenocysteine tRNAs (TCA)	5
Number of bp in repeat regions (Mb)	41.5
Percentage of Repeat region in genome (%)	15.71

Supplementary Table 3. Repetitive elements of *A. avenae* genome.

Feature	Number of elements	Length occupied	Percentage of sequence
LINEs	2023	1131182 bp	0.43%
L3/CR1	1369	685448 bp	0.26%
LTR elements:	5965	3459712 bp	1.31%
DNA elements	27636	5837268 bp	2.21%
Unclassified	139721	26626410 bp	10.09%
Total interspersed repeats		37054572 bp	14.04%
Small RNA	414	100809 bp	0.04%
Satellites	1885	411036 bp	0.16%
Simple repeats	30797	3913828 bp	1.48%
Low complexity	8631	439649 bp	0.17%

Total length: 264011243 bp (223441246 bp excl N/X-runs)
GC level: 49.72 %
Bases masked: 41477910 bp (15.71 %)

Supplementary Table 4. Pfam domain comparisons among *A. avenae* (Aa), *C. elegans* (Ce), fruit fly (Dm), Sea Urchin (Sp) and human (Hs).

Process	Domain	PFAM no.	Aa	Ce	Dm	Sp	Hs
Cell cycle control	Cyclin_N	PF00134	14	7	11	15(17)	21
	Cyclin_C	PF02984	4	5	4	7(8)	12
	E2F_TDP	PF02319	7	4	3	3(5)	11
	RB_A	PF01858	1	1	2	2	3
	RB_B	PF01857	1	1	2	2	3
	Cullin	PF00888	25	7	8	7	9
	Skp1	PF01466	117	21	5	1	3
Histone metabolism	Histone	PF00125	51	0	8	49	75
	Linker histone	PF00538	8	0	2	5	8
	Nucleo-plasmin	PF03066	0	0	2	2	5
	NAP	PF00956	5	2	4	2	24
	HDAC	PF00850	15	8	5	8	11
	DOT1	PF08123	4	6	1	1	1
RNA metabolism	RRM_1	PF00076	167	99	126	140(178)	245
	TUDOR	PF00567	19	8	15	15	13
	DEAD	PF00270	103	65	56	93(125)	78
	LSM	PF01423	21	18	17	17	21
	KH-1	PF00013	47	28	28	28(31)	36
	DSRM	PF00035	9	13	14	14(15)	21
	3'-5'-Exo-nuclease	PF01612	23	9	5	13(15)	5

	Exonuc_X-T	PF00929	17	10	7	9(11)	15
Apoptosis	Caspase	PF00656	10	4	7	31(33)	14
	BIR	PF00653	2	1	4	4(7)	8
	Bcl-2	PF00452	0	1	2	10	11
	TNFR_c6	PF00020	2	1 (no DD)	1(no DD)	8(9)	8
	NACHT	PF05729	2	1	1	129(145)	18
	NB-ARC	PF00931	4	1	1	3	1
	DEATH	PF00531	3	6	9	47(101)	30
	DED	PF01335	0	0	1	4(5)	7
	CARD	PF00619	1	0	1	5(10)	20

Supplementary Table 5. Summary of sequencing reads for *A. avenae* transcriptomes under the conditions including 100% (R.H.100), 97% (R.H.97), 85% (R.H.85), 40% (R.H.40), and 0% (R.H.0) relative humidity.

Sample name	Read number
R.H.100-1	53M
R.H.100-2	28M
R.H.100-3	60M
R.H.97-1	33M
R.H.97-2	29M
R.H.97-3	28M
R.H.85-1	30M
R.H.85-2	29M
R.H.85-3	24M
R.H.40-1	40M
R.H.40-2	27M
R.H.40-3	30M
R.H.0-1	31M
R.H.0-2	24M
R.H.0-3	24M

Supplementary Table 6. Training accuracy of gene prediction program AUGUSTUS.

	Sensitivity	Specificity
Nucleotide level	0.961	0.807
Exon level	0.846	0.689
Gene level	0.45	0.29

Supplementary Table 7. Comparison of orthologous genes between *A. avenae* and clade IV species

	Total size of assembled genome (Mb)	Gene models	Gene density (genes per Mb)	proteins in 8 spp. shared families (percentage)	proteins in other multi-gene families (percentage)	proteins in lineage-specific gene families (percentage)	singleton proteins (percentage)
A. avenae	264.8	43192	163	8532 (19.8%)	14045 (32.5%)	17637 (40.8%)	2978 (6.9%)
Steinernema carpocapsae	86.1	31937	371	6882 (21.5%)	9081 (28.4%)	8724 (27.3%)	7250 (22.7%)
Meloidogyne hapla	53	14419	272	4658 (32.3%)	8152 (56.5%)	743 (5.2%)	866 (6.0%)
Globodera pallida	123.6	16403	133	5533 (33.7%)	5202 (31.7%)	4166 (25.4%)	1502 (9.2%)
Meloidogyne incognita (a)	183.5	43718	238	13840 (31.7%)	23564 (53.9%)	3756 (8.6%)	2558 (5.9%)
Meloidogyne incognita (b)	122	21830	179	7867 (36.0%)	11912 (54.6%)	805 (3.7%)	1246 (5.7%)
Bursaphelenchus xylophilus	74.6	17704	237	4975 (28.1%)	6328 (35.7%)	3331 (18.8%)	3070 (17.3%)
Panagrolaimus superbus	76.7	19663	256	5730 (29.1%)	6469 (32.9%)	3890 (19.8%)	3574 (18.2%)

(a) PRJEB8714, population Morelos
(b) PRJNA340324, W1 strain

Supplementary Table 8. List of accession numbers of late embryogenesis abundant protein (LEA) group 3 protein sequences referred in this study.

Organism	Accession
Caenorhabditis remanei	XP_003116340.1
Caenorhabditis elegans	NP_001256173.1
Caenorhabditis elegans	NP_001256166.1
Zea mays	NP_001105298.1
Triticum aestivum	AAN74639.1
Arabidopsis thaliana	BAA11017.1
Medicago truncatula	XP_003590405.1
Aphelenchus avenae	AAL18843.1

Supplementary Table 9. Differential gene expression of late embryogenesis abundant proteins (LEA proteins) in *A. avenae* genome (n=3 biologically independent samples).

Gene	Start	End	Strand	Multi exon	Sample fresh	Sample RH97	Status	FPKM _1	FPKM _2	Log2(fold _change)	Significance
AAVH_01701	1863572	1864170	-	y	fr	97	OK	191.066	1940.22	3.34	yes
AAVH_05549	277234	278733	+	y	fr	97	OK	212.786	1529.66	2.85	yes
AAVH_06103	655211	655792	+	y	fr	97	OK	248.698	1967.66	2.98	yes

AAVH_06033	380295	380628	-	y	fr	97	OK	49.5954	11224.8	7.82	yes
AAVH_07541	551009	552580	-	y	fr	97	OK	98.6388	1534.79	3.96	yes
AAVH_09857	218089	219926	-	y	fr	97	OK	60.7841	1897.21	4.96	yes
AAVH_10501	216112	220776	-	y	fr	97	OK	7.12522	985.353	7.11	yes
AAVH_11783	186801	188168	+	y	fr	97	OK	43.9527	2686.4	5.93	yes
AAVH_16491	131993	132402	-	y	fr	97	OK	25.5865	2002.37	6.29	yes
AAVH_16492	134263	134544	-	y	fr	97	OK	4.37686	340.903	6.28	yes
AAVH_26283	56953	57891	-	n	fr	97	NOTEST	0	0	0	no
AAVH_30700	6624	8070	+	y	fr	97	OK	84.1427	6259.72	6.22	yes
AAVH_35531	1247	1568	-	y	fr	97	OK	4.29734	2231.94	9.02	yes
AAVH_41847	428	1915	+	y	fr	97	OK	74.9894	1784.58	4.57	yes
AAVH_42413	857	2225	+	y	fr	97	OK	35.3324	1854.58	5.71	yes

Supplementary Table 10. Summary of kinase families in *A. avenae*, *C. elegans*, *C. briggsae*, *B. malayi*, and *H. sapiens*.

**Kinome**	***A. avenae***	***C. elegans***	***C. briggsae***	***B. malayi***	***H. sapiens***
EPKs					
AGC	517	35	46	22	84
CAMK	81	63	69	41	98
CK1	46	91	77	31	12
CMGC	12	56	60	33	70
RGC	43	27	24	4	5
STE	25	35	27	27	61
TK	35	96	73	35	93
TKL	8	22	21	12	55
**Total**	767	425	397	205	478
APKs					
PIKK	10	5	4	5	6
Alpha	3	1	1	1	6
PDHK	1	1	1	1	5
RIO	5	3	3	3	3
**Total**	19	10	9	10	20

Supplementary References

1. Grabherr, M.G. *et al*. Trinity: reconstructing a full-length transcriptome without a genome from RNA-Seq data. *Nat Biotechnol*. **29**, 644 (2011).

2. Luo, R. *et al*. SOAPdenovo2: an empirically improved memory-efficient short-read de novo assembler. *GigaScience* **1**, 1-6. (2012).

3. Parra, G., Bradnam, & K., Korf, I. CEGMA: a pipeline to accurately annotate core genes in eukaryotic genomes. *Bioinformatics* **23**, 1061–1067 (2007).

4. Ali, M.R., Yamagughi, Y., & Ishibashi, N. RAPD and PCR-RFLP analysis on genetic diversity of *Aphelenchus avenae* isolates from Kyushu and some other districts of Japan. *Japanese Journal of Nematology* **29**, 24–34 (1999).

5. Li, H. *et al*. 1000 Genome Project Data Processing Subgroup. The Sequence alignment/map (SAM) format and SAMtools. *Bioinformatics* **25**, 2078-2079 (2009).

6. Ye, J. *et al*. WEGO: a web toll for plotting GO annotations. *Nucleic. Acids Res*. **34**, 293-297 (2006).

7. Ye. J. *et al*. WEGO 2.0: a web tool for analyzing and plotting GO annotations, 2018 update. *Nucleic. Acids Res*. **46**, W71-W75 (2018).

8. Serra, L. *et al*. Hybrid assembly of the Genome of the Entomopathogenic nematode *Steinernema carpocapsae* identifies the X-chromosome. *G3* **9**, 2687-2697 (2019).

9. Opperman, C. H. *et al*. Sequence and genetic map of *Meloidogyne hapla*: A compact nematode genome for plant parasitism. *Proc. Natl. Acad. Sci. USA*. **105**, 14802-14807 (2008).

10. Cotton, J. A. *et al*. The genome and life-stage specific transcriptomes of *Globodera pallida* elucidate key aspects of plant parasitism by a cyst nematode. *Genome Biology* **15**, R43 (2014).

11. Blanc-Mathieu, R. *et al*. Hybridization and polyploidy enable genomic plasticity without sex in the most devastating plant-parasitic nematodes. *PLoS Genetics* **13**, e1006777 (2017).

12. Szitenberg, A. *et al*. Comparative genomics of apomictic root-knot nematodes: hybridization, ploidy, and dynamic genome change. *Genome Biol. Evol*. **9**, 2844-2861 (2017).

13. Kikuchi, T. *et al*. Genomic insights into the origin of parasitism in the emerging plant pathogen *Bursaphelenchus xylophilus*. *PLoS Pathogen* **7**, e1002219 (2011).

14. Schiffer, P. H. *et al*. Signatures of the evolution of parthenogenesis and cryptobiosis in the genomes of Panagrolaimid nematodes. *iScience* **21**, 587-602 (2019).

15. Emms, D. M., & Kelly, S. OrthoFinder: solving fundamental biases in whole genome comparisons dramatically improves orthogroup inference accuracy. *Genome Biology* **16**, 157 (2015).

16. Emms, D. M., & Kelly, S. OrthoFinder: phylogenetic orthology inference for comparative genomics. *Genome Biology* **20**, 238 (2019).

17. Sutton, R.E., & Boothroyd, J.C. Evidence for trans splicing in trypanosomes. *Cell* **47**, 527–535 (1986).

18. Krause, M., & Hirsh, D. A trans-spliced leader sequence on actin mRNA in *C. elegans*. *Cell* **49**, 753–761 (1987).

19. Vandenberghe, A.E., Meedel, T.H., & Hastings, K.E. mRNA 5'-leader trans-splicing in the chordates. *Genes Dev*. **15**, 294–303 (2001).

20. Allen, M.A., Hillier, L., Waterston, R.H. & Blumenthal, T. A global analysis of trans-splicing in *C. elegans*. *Genome Res*. **21**, 255-264 (2011).

21. Dosztányi, Z., Csizmók, V., Tompa, P., & Simon, I. IUPred: web server for the prediction of intrinsically unstructured regions of proteins based on estimated energy content. *Bioinformatics* **21**, 3433-3434 (2005).

22. Goyal, K., Walton, L.J., & Tunnacliffe, A. LEA proteins prevent protein aggregation due to water stress. *Biochem. J*. **388**, 151-159 (2005).

23. Kovacs, D., Kalmar, E., Torok, Z., & Tompa, P. Chaperone activity of ERD10 and ERD14, two disordered stress-related plant proteins. *Plant Physiol* **147**, 381–390 (2008).

24. Dure, L. A repeating 11-mer amino acid motif and plant desiccation. *The Plant Journal* **3**, 363-369 (1993).

25. Browne, J., Tunnacliffe, A., & Burnell, A. Anhydrobiosis: plant desiccation gene found in a nematode. *Nature* **416**, 38 (2002).

26. Chakrabortee, S. *et al*. Hydrophilic protein associated with desiccation tolerance exhibits broad protein stabilization function. *Proc. Natl. Acad. Sci. USA*. **104**, 18073-18078 (2007).

27. Larkin, M. A. *et al*. Clustal W and Clustal X version 2.0. *Bioinformatics* **23**, 2947-2948 (2007).

28. Zhang, H., Gao, S., Lercher, M. J., Hu, S., & Chen, W. EvolView, an online tool for visualizing, annotating and managing phylogenetic trees. *Nucleic. Acids Res.* **W1**, W569-W572 (2012).

29. Söding, J. Protein homology detection by HMM-HMM comparison. *Bioinformatics* **21**, 951–960 (2005).

30. Remmert, M. et al. HHblits: lightning-fast iterative protein sequence searching by HMM-HMM alignment. *Nat. Methods* **9**, 173–175 (2012).

31. Uéda, K. *et al*. Molecular cloning of cDNA encoding an unrecognized component of amyloid in Alzheimer disease. *Proc. Natl. Acad. Sci. USA*. **90**, 11282- 11286 (1993).

32. Cookson, M. R. Alpha-synuclein and neuronal cell death. *Mol. Neurodegener* **4**, 9. (2009).

33. Morrow, G., Samson, M., Michaud, S., & Tanguay, R. M. Overexpression of the small mitochondrial Hsp22 extends *Drosophila* life span and increases resistance to oxidative stress. *FASEB J*. **18**, 598–599 (2004).

34. Walker, G.A., & Lithgow, G.J. Lifespan extension in *C. elegans* by a molecular chaperone dependent upon insulin-like signals. *Aging Cell* **2**, 131-139 (2003).

35. Miranda-Saavedra, D., & Barton, G.J. Classification and functional annotation of eukaryotic protein kinases. *Proteins* **68**, 893-914 (2007).

36. Ispolatov, I., Yuryev, A., Mazo, I., & Maslov, S. Binding properties and evolution of homodimers in protein-protein interaction networks. *Nucleic Acids Res*. **33**, 3629-3635 (2005).
