# Supplementary material for: The Aphelenchus avenae genome highlights evolutionary adaptation to desiccation
Source: Commun Biol. 2021 Oct 28;4:1232. doi: 10.1038/s42003-021-02778-8 (PMC8553787; doi:10.1038/s42003-021-02778-8)
Supplement: Supplementary file 3 — Description of Additional Supplementary Files [file 42003_2021_2778_MOESM3_ESM.pdf]

Description of Additional Supplementary Files

File name: Supplementary Data 1-7
Description:

Supplementary Data 1: Summary of Pfam domains in A. avenae genome.

Supplementary Data 2: List of gene models predicted to be in possible operons.

Supplementary Data 3: List of collinear duplicated gene models in A. avenae genome.

Supplementary Data 4: List of identified A. avenae intrinsically disordered proteins (IDP) with their differential gene expression values (n=3 biologically independent samples).

Supplementary Data 5: List of species-specific IDPs in A. avenae genome.

Supplementary Data 6: List of 70 kilodalton heat shock proteins (Hsp70) with their differential gene expression values (n=3 biologically independent samples).

Supplementary Data 7: List of differentially expressed kinases (n=3 biologically independent samples).
